# Metagenomic Analysis Suggests Modern Freshwater Microbialites Harbor a Distinct Core Microbial Community

**DOI:** 10.3389/fmicb.2015.01531

**Published:** 2016-01-28

**Authors:** Richard Allen White, Amy M. Chan, Gregory S. Gavelis, Brian S. Leander, Allyson L. Brady, Gregory F. Slater, Darlene S. S. Lim, Curtis A. Suttle

**Affiliations:** ^1^Department of Microbiology and Immunology, University of British Columbia, VancouverBC, Canada; ^2^Department of Earth, Ocean and Atmospheric Sciences, University of British Columbia, VancouverBC, Canada; ^3^Department of Zoology, University of British Columbia, VancouverBC, Canada; ^4^Department of Botany, University of British Columbia, VancouverBC, Canada; ^5^School of Geography and Earth Sciences, McMaster University, HamiltonON, Canada; ^6^Bay Area Environmental Institute, PetalumaCA, USA; ^7^NASA Ames Research Center, Moffett FieldCA, USA; ^8^Canadian Institute for Advanced Research, TorontoON, Canada

**Keywords:** microbialites, Pavilion Lake, metagenomics, thrombolites, metabolic potential

## Abstract

Modern microbialites are complex microbial communities that interface with abiotic factors to form carbonate-rich organosedimentary structures whose ancestors provide the earliest evidence of life. Past studies primarily on marine microbialites have inventoried diverse taxa and metabolic pathways, but it is unclear which of these are members of the microbialite community and which are introduced from adjacent environments. Here we control for these factors by sampling the surrounding water and nearby sediment, in addition to the microbialites and use a metagenomics approach to interrogate the microbial community. Our findings suggest that the Pavilion Lake microbialite community profile, metabolic potential and pathway distributions are distinct from those in the neighboring sediments and water. Based on RefSeq classification, members of the *Proteobacteria* (e.g., alpha and delta classes) were the dominant taxa in the microbialites, and possessed novel functional guilds associated with the metabolism of heavy metals, antibiotic resistance, primary alcohol biosynthesis and urea metabolism; the latter may help drive biomineralization. Urea metabolism within Pavilion Lake microbialites is a feature not previously associated in other microbialites. The microbialite communities were also significantly enriched for cyanobacteria and acidobacteria, which likely play an important role in biomineralization. Additional findings suggest that Pavilion Lake microbialites are under viral selection as genes associated with viral infection (e.g CRISPR-Cas, phage shock and phage excision) are abundant within the microbialite metagenomes. The morphology of Pavilion Lake microbialites changes dramatically with depth; yet, metagenomic data did not vary significantly by morphology or depth, indicating that microbialite morphology is altered by other factors, perhaps transcriptional differences or abiotic conditions. This work provides a comprehensive metagenomic perspective of the interactions and differences between microbialites and their surrounding environment, and reveals the distinct nature of these complex communities.

## Introduction

Microbialites are a specialized group of microbial mats that lithify carbonate-rich structures, and include thrombolites that consist of structures with unlaminated clots and stromatolites that have laminated layers (Burne and Moore, 1987; Perry et al., 2007). Fossil evidence points to microbialites being representative of the oldest known persistent ecosystems (Grotzinger and Knoll, 1999; Schopf, 2006). They are an unparalleled system in which to investigate biochemical cycling that may be representative of the earliest known complex microbial communities (Dupraz et al., 2009).

Microbialites are globally distributed, and can be found in marine (Reid et al., 2000; Burns et al., 2004; Khodadad and Foster, 2012; Mobberley et al., 2013), freshwater (Ferris et al., 1997; Laval et al., 2000; Gischler et al., 2008; Breitbart et al., 2009; Couradeau et al., 2011), hypersaline (Allen et al., 2009; Goh et al., 2009), hot spring (Bosak et al., 2012), and remnant mining (Power et al., 2011) environments. To a lesser extent, they have also colonized terrestrial environments, such as landfill soils (Maliva et al., 2000) and caves (Lundberg and McFarlane, 2011). These microbial communities thrive at the intersection of abiotic and biotic factors that promote organosedimentation (Dupraz and Visscher, 2005; Dupraz et al., 2009).

A host of biological factors are favorable to microbialite formation, such as the presence of exopolysaccaride (EPS)-rich cyanobacterial mats, which serve as a location of mineral nucleation and provide a heterotrophic microenvironment favorable for organomineralization via dissimilatory sulfate reduction (Dupraz and Visscher, 2005; Dupraz et al., 2009). Cyanobacterial photosynthetic activity increases pH in the surrounding geochemical environment, promoting precipitation by raising the calcium carbonate saturation index critical to the formation process of microbialites (e.g., Merz, 1992; Dupraz et al., 2009). Microbialites have been purported to form via carbonate precipitation by the benthic community, as well as by trapping detritus from sediment and the overlying water column (Burne and Moore, 1987; Dupraz and Visscher, 2005).

Despite the apparent reliance of microbialites on biotic input from the surrounding environment, there is currently a scarcity of data comparing microbialite communities with those of the sediments or water. Such a comparison allows for the identification of microbialite-specific components that may not be obvious when examining microbialite communities in isolation. Exploring the genetic differences between microbialite communities and those in the surrounding habitats requires identifying the relative abundance of taxa and their metabolic potential. To avoid distorting these ratios, DNA was extracted and unlike in other metagenomic studies of microbialites (Breitbart et al., 2009), sequenced without amplification.

Many studies have focused on examining the abundance and diversity of freshwater microbialites using 16S rDNA sequencing but few metagenomic studies exist. Diversity studies using 16S rDNA amplicons on freshwater microbialites include Lake Van (López-García et al., 2005), Lake Alchichica (Couradeau et al., 2011), Cuatro Ciénegas (Centeno et al., 2012), Pavilion Lake (Chan et al., 2014; Russell et al., 2014), and Ruidera Pools (Santos et al., 2010). While 16S rDNA sequencing is able to obtain the relative abundance of taxa and diversity of taxa; it is unable to capture the metabolic potential or the functional gene abundance of an ecosystem. Metabolic and functional potential obtained by metagenomics allow for functional gene inventories which can be used as databases for further investigations using other omics (e.g., metaproteomics) (Cantarel et al., 2011). Prior metagenomics studies on microbialites have focused primarily on marine environments (Khodadad and Foster, 2012; Mobberley et al., 2013; Ruvindy et al., 2015) with the exception of one tropical freshwater system (Breitbart et al., 2009) and one subarctic abandoned open pit mine (White III et al., 2015). In this study, we sequenced total genomic DNA from cold temperate freshwater microbialites, as well as from the nearby sediment and water, to identify constituent taxa and infer their metabolic functional potential.

**FIGURE 1:**
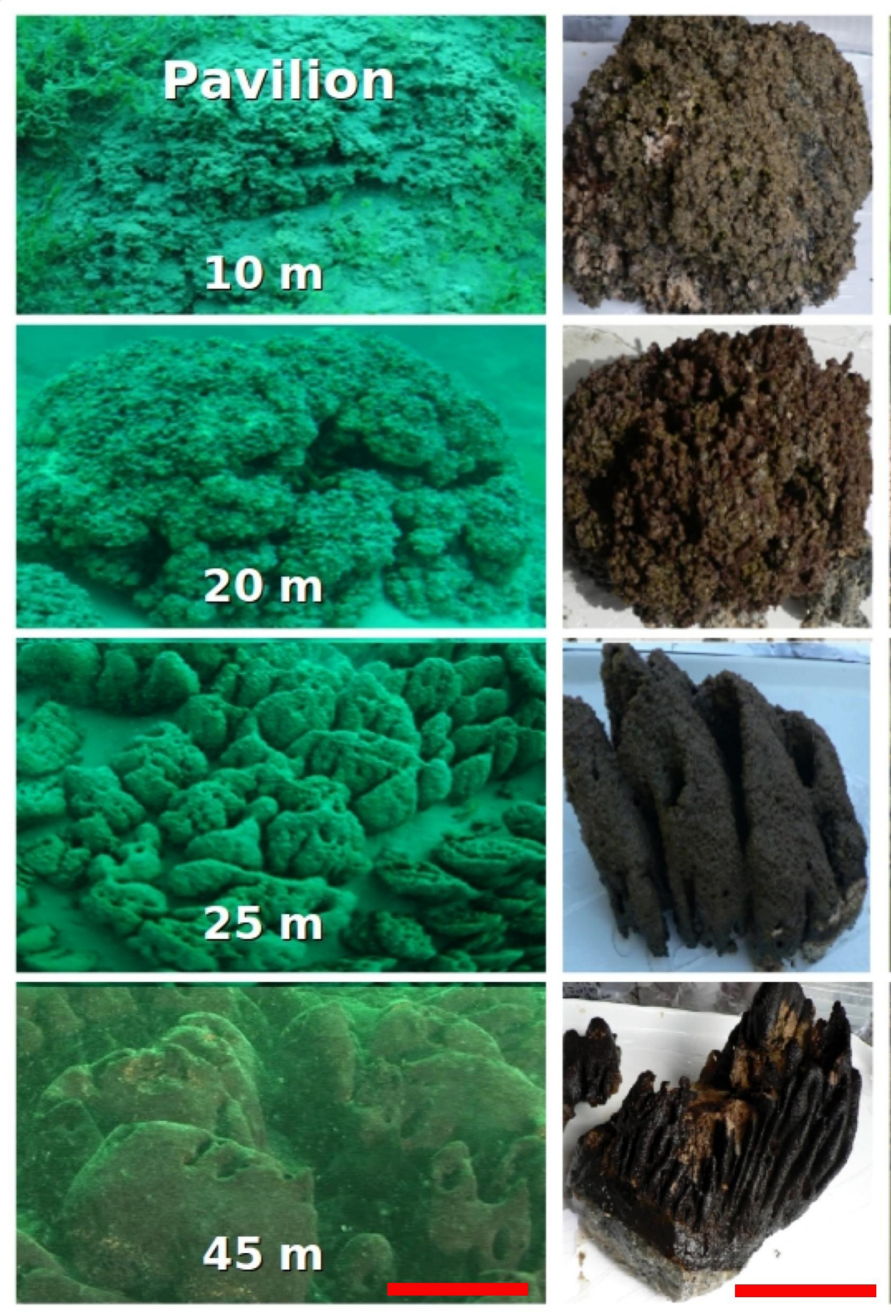
**Pavilion Lake microbialite morphology as a function of sampling depth in meters.** The scale bar is ∼1 m **(left)** and ∼15 cm **(right)**.

Sampling was conducted in Pavilion Lake, in southeastern British Columbia, Canada (50.8°N, 121.7°W). The lake is dimictic, circumneutral (median pH 8.3; mean calcium carbonate, 182 mg L^-1^), and oligotrophic (mean total phosphorus, 3.3 μg L^-1^). Further limnological details of Pavilion Lake are given in Lim et al. (2009). The microbialites are primarily calcite thrombolites, covered in a thin (∼5 mm) microbial mat, that change in morphotype with depth; at ∼10 m they resemble shallow domes, at ∼20 m they resemble cabbage heads, at 25 m they consist of conical outcroppings, and at deeper depths they possess mound structures (**Figure 1**; Laval et al., 2000). However, whether morphological changes in the microbialites are associated with changes in community structure or metabolic potential is not well constrained.

Data suggest that photosynthetically induced alkalinization is a major driver of recent carbonate precipitation in shallow Pavilion Lake microbialites (Brady et al., 2010). Two recent 16S studies of Pavilion Lake microbialites indicated that cyanobacteria, including members of the genera *Acaryochloris*, *Leptolyngbya*, *Microcoleus*, and *Pseudanabaena*, are dominant oxygenic photoautotrophic members (Chan et al., 2014; Russell et al., 2014). Moreover, elevated O_2_ concentrations, pH and δ^13^C carbonate values within surface microbial mats and cyanobacterial rich nodules from microbialites at < 20 m depth indicate photosynthetic influence on carbonate that is being precipitated (Brady et al., 2010, 2014). Whether biomineralization in this system is strictly a photosynthetic process or a mixture of heterotrophic and photosynthetic processes remains unconstrained.

In addition to challenges associated with elucidating the role of bacteria and eukarya in the formation of microbialite structures, the role of viruses in microbialite communities has remained elusive. Viruses are the most prevalent “organisms” on Earth, with an estimated 10^30^ viruses in the ocean (Suttle, 2005). Through cell lysis, they play a role in carbon cycling on a global scale (Suttle, 2005). Metagenomic data for the viral fraction have been published for marine (e.g., Highbourne Cay) and freshwater microbialites (e.g., Cuatro Cienegás) (Desnues et al., 2008). However, because the surrounding water and sediments were not sampled, it is unclear whether the viral taxa were specifically associated with the microbialites, or were derived from the surrounding environments.

In this contribution, a metagenomic approach was used to uncover the metabolic potential that is specifically associated with microbialites. We examine the novel metabolic potential associated with Pavilion Lake microbialites and investigate whether metabolic potential, not solely taxa, changes as a function of microbialite morphology. We also explore whether heterotrophic or phototrophic pathways dominate the microbialite functional metabolic potential in association with carbonate precipitation, and examine virus-host whole community interactions. As well, we address the question of whether the microbialite communities are distinct from those found in other microbialite systems and in the adjacent water and sediment.

## Materials and Methods

### Sample Collection

Samples were collected from Pavilion Lake (50.86°N, 121.74°W) during the summers of 2010 and 2011. Triplicate representative microbialites (∼10 kg), were recovered from each collection site (Lim et al., 2011) either by SCUBA divers along a transect [Three Poles (TP) site: 10, 20, 25 m] or by manned DeepWorker submersible (Deep mound site: 45 m; 2010) (Nuytco Research Ltd., North Vancouver, BC, Canada). Diver collected microbialites were placed into separate plastic bags and brought to the surface, whereas DeepWorker samples were collected by a robotic arm and placed into a basket before transport to the surface.

Sediment samples, adjacent to the microbialites (∼20 g of the surface layer; 10, 20, 25 m depths; 2011) were collected into sterile bottles at the same time by divers. At the lake surface, microbialite and sediment samples were immediately placed into insulated containers filled with cold lakewater to maintain *in situ* temperatures until samples were processed. At the field lab, each microbialite was weighed, documented and apportioned for molecular analysis. Replicate sediment samples were pelleted by centrifugation (5000 × *g*). The overlying lakewater was removed and the sediment pellets flash frozen in liquid nitrogen and transported back to the lab in a liquid nitrogen vapor shipper for downstream processing.

Water adjacent to the microbialites (∼100 L) was collected from each depth using a Niskin water sampler (2010) or a diver guided hose at the collection site that was connected to a piston-pump in a boat (2011). Surface water samples (∼1 m depth) were collected using a submersible pump. Each water sample was filtered in series through 120-μm pore-size Nitex^®^ screening to remove large plankton, and 1.2-μm pore-size glass-fiber, followed by 0.45 and 0.22-μm pore-size Durapore polyvinylidene difluoride (PVDF) filters (Millipore, Bedford, MA, USA) (Suttle et al., 1991). Filters were frozen in the field and transported back to the lab in a liquid nitrogen vapor shipper.

### DNA Extraction

To sample the microbialite associated microbial communities, a sterile razor blade was used to scrape off 3 to 10 mm (∼5 g) across the surface of three morphologically similar microbialites collected at each depth. DNA was extracted on-site using a PowerMax^®^ Soil DNA Isolation Kit (Mobio, Carlsbad, CA, USA) then flash frozen in liquid nitrogen. Replicate microbialite scrapings were placed into sterile jars and frozen on-site in liquid nitrogen. Frozen samples were transported back to the lab in a liquid nitrogen vapor shipper and stored at –80°C until needed. DNA from frozen samples were extracted using cetyl trimethyl ammonium bromide (CTAB; Untergasser, 2008).

To ascertain the microbial community from the water column (size fraction between 0.2 and 120-μm), DNA was extracted from half of each glass-fiber, 0.45 and 0.22-μm pore-size filter using a PowerWater^®^ DNA Isolation Kit (Mobio, Carlsbad, CA, USA). DNA was extracted from the other half of each filter using the CTAB method (Untergasser, 2008).

Sediment DNA was extracted from triplicate subsamples (∼5 g) using a PowerMax^®^ Soil DNA Isolation Kit (Mobio, Carlsbad, CA, USA). Replicate sediment pellets were also extracted using the CTAB method. Two DNA extraction methods were employed for all samples to minimize extraction biases.

DNA concentrations were determined on-site using a Nanodrop-3300 micro-fluorospectrometer and the Quant-iT^TM^ PicoGreen^®^ dsDNA Assay Kit (ThermoFisher, Wilmington, DE, USA). Nucleic acid quality was determined by absorbance (260/280 and 260/230) using a Nanodrop-1000 (ThermoFisher, Wilmington, DE). CTAB and MoBio DNA extractions were pooled (50:50) by equivalent DNA to reduce extraction bias and then used for library construction.

### Metagenomic Library Preparation: 454 FLX Titanium and Illumina HiSeq/MiSeq

Libraries for 454 FLX Titanium sequencing were constructed using random DNA shearing with a Bioruptor (Diagenode Denville, NJ, USA). Fragments were polished and blunt-end ligated (NEBNext DNA Library Prep Kit, New England Biolabs, Ipswich, MA, USA) to in-house Multiplex Identifier barcode oligos (IDT, Coralville, IA, USA), with small fragments removed by magnetic beads (Beckman Coulter, Danvers, MA, USA). The libraries were quantified using a digital PCR quantified standard curve (White III et al., 2009), diluted, and pooled for 454 pyrosequencing with Titanium chemistry (The Centre for Applied Genomics, SickKids Hospital, Toronto, ON, Canada).

For Illumina library construction, DNA was sheared by ultrasonication (Covaris M220 series, Woburn, MA, USA), and the fragments end-paired, A-tailed (NxSeq DNA Sample Prep Kit, Lucigen, Middleton, WI, USA) and ligated to TruSeq adapters (IDT, Coralville, IA, USA); small fragments were removed twice using magnetic beads (Beckman Coulter, Danvers, MA). The resulting libraries were pooled, and sequenced using both 250 bp and 100 bp paired-end sequencing on the MiSeq (UCLA Genotyping & Sequencing Core, Los Angeles, CA, USA) and HiSeq (McGill University and Génome Québec Innovation Centre, Montreal, QC, Canada) platforms, respectively.

### Metagenomic Data Assembly and Analysis

The raw sequencing data were processed as follows. For the 454 data, the raw SFF files were converted to FASTQ format and binned by molecular barcode (MID) using a custom Perl script. Barcodes were removed by Tagcleaner (Schmieder et al., 2010) and sequences cleaned for low quality and homopolymers using PRINSEQ (Schmieder and Edwards, 2011). The Illumina data were extracted and demultiplexed using the CASAVA pipeline v1.8 (Illumina, San Diego, CA, USA), and the PhiX spike-in used for sequencing quality control was screened using Bowtie2 (version 2.1.0; Langmead and Salzberg, 2012) then removed using Picard tools^1^ (version 1.90; White III and Suttle, 2013; White III et al., 2013a,b).

The resulting 454 FLX titanium reads, Illumina overlapping merged reads and Illumina non-overlapping reads from replicate libraries were combined and assembled (kmer size: 39) using the Ray DeNovo assembler (Boisvert et al., 2010, 2012). Illumina sequencing compensates for the error-prone homopolymers of 454, while 454 compensates for Illumina’s GC bias and substitution errors (Bentley et al., 2008). A hybrid of the two technologies provides a lower chance of obtaining the same sequencing error and results in higher quality assembly at lower cost (Aury et al., 2008). In total, 446 Mbp and 17 Mbp of assembled contigs were obtained from the microbialites and filters (**Table 1**), respectively. Surface (∼1 m) and 10 m water metagenomic reads were pooled at assembly step to yield >15 k contigs for further comparison. Sediment metagenomic data (84 Mbp in total) resulted in a low numerical (<15 k) yield of contigs; hence, the unassembled paired-end reads were extended for overlap and pooled with unextended reads for further analysis (**Table 1**). Metagenomic rapid annotations using MG-RAST were used for contig annotation (Meyer et al., 2008). MG-RAST annotation of the contigs used BLAT (BLAST-like alignment tool) annotations based on hierarchical classification against SEED subsystems^2^ and RefSeq databases^3^ with a minimum *E*-value cutoff of 10^-5^, a minimum percent identity cutoff of 60%, and a minimum alignment length cutoff of 50 base pairs.

**Table 1:** Pavilion Lake metagenome assembly statistics using Ray Meta DeNovo Assembler.

Depth	10 m	20 m	25 m	45 m	Sfc^∗∗^	20 m	25 m	10 m	20 m	25 m
No. Contig or Read	510415	881449	499134	179215	43909	19977	32086	120680	544185	94150
Total Length (Bp)	115196707	178186326	111159198	41483863	8537385	3836213	5930888	12560841	61059259	10421166
N50 > 500bp	647	752	717	668	649	734	608	(–)	(–)	(–)
Avg > 500bp	721	783	760	736	776	753	644	(–)	(–)	(–)
Med > 500bp	571	633	616	621	524	670	575	(–)	(–)	(–)
Largest (Bp)	3882	6079	2965	1964	7125	4003	1880	190	190	190
G+C%	60.8	57.9	60.1	60.3	47	52.8	52.6	59.6	57.7	52.2


*Sediment data were not assembled. Surface water (Sfc) (1 and 10 m samples) of water pooled at the assembly step.*

*^∗^Not assembled reads only*

*^∗∗^Sfc, surface water filter (∼1 to 10 m)*

*(–) not available.*

MetaCyc^4^ annotations were provided by MetaPathways, a modular pipeline for gene prediction and annotation that uses pathway tools and the MetaCyc database to construct environmental pathway/genome databases (ePGBDs; Konwar et al., 2013). Metapathways using the LAST (local alignment search tool) for annotations of ORFs with a minimum of 180 bp and minimum alignment length cutoff of 50 bp (Kiełbasa et al., 2011). MetaCyc pathway comparison Venn diagrams were based on normalized pathway size and number of open reading frames (ORFs) associated with each pathway using R, then plotted using ggplot2 (Wickham, 2009).

Statistical analysis was completed using statistical analysis of metagenomic profiles (STAMP) and R (Parks and Beiko, 2010; R Development Core Team, 2015). STAMP and R were used to parse MG-RAST data for RefSeq (class level) and SEED subsystems (level I to function) results. The STAMP ANOVAs (including Principal Component Analysis, PCA) were completed using multiple groups, *post-hoc* tests (Tukey-Kramer at 0.95), an effect size (Eta-squared) and multiple test correction using Benjamini-Hochberg FDR (false discovery rate) procedure. The RefSeq and SEED classifications were normalized for each sample using count-relative abundances and total ORFs obtained per metagenome. PCA for the normalized RefSeq and SEED classifications used R libraries Ecodist (Dissimilarity-based functions for ecological analysis), and pvclust (Hierarchical Clustering with *P*-values via Multiscale Bootstrap Resampling) using ward clustering and the Bray-Curtis distance matrix at a thousand replicates (Suzuki and Shimodaira, 2006). The PCA for the normalized RefSeq and SEED classifications were plotted using R libraries ggplot2 and a dotplot was created using R libraries Reshape2, using the melt function, then plotted using ggplot2 (Wickham, 2009).

### Metagenomic Data Depositing

All the data used in this study is freely available from MG-RAST^5^. The data is deposited in the project name Pavilion Lake surrounding environment as PLsfcFil (ID 4532785.3), PL20Fil (ID 4532783.3), PL45Fil (ID 4532784.3), PLMB10 (ID 4532771.3), PLMB20 (ID 4532772.3), PLMB25 (ID 4532774.3), PLMB45 (ID 4532775.3), PL10Sed (ID 4526738.3), PL20Sed (ID 4526739.3), and PL25Sed (ID 4526740.3).

## Results and Discussion

### Microbialite Communities Differ from the Surrounding Environment Communities

**FIGURE 2:**
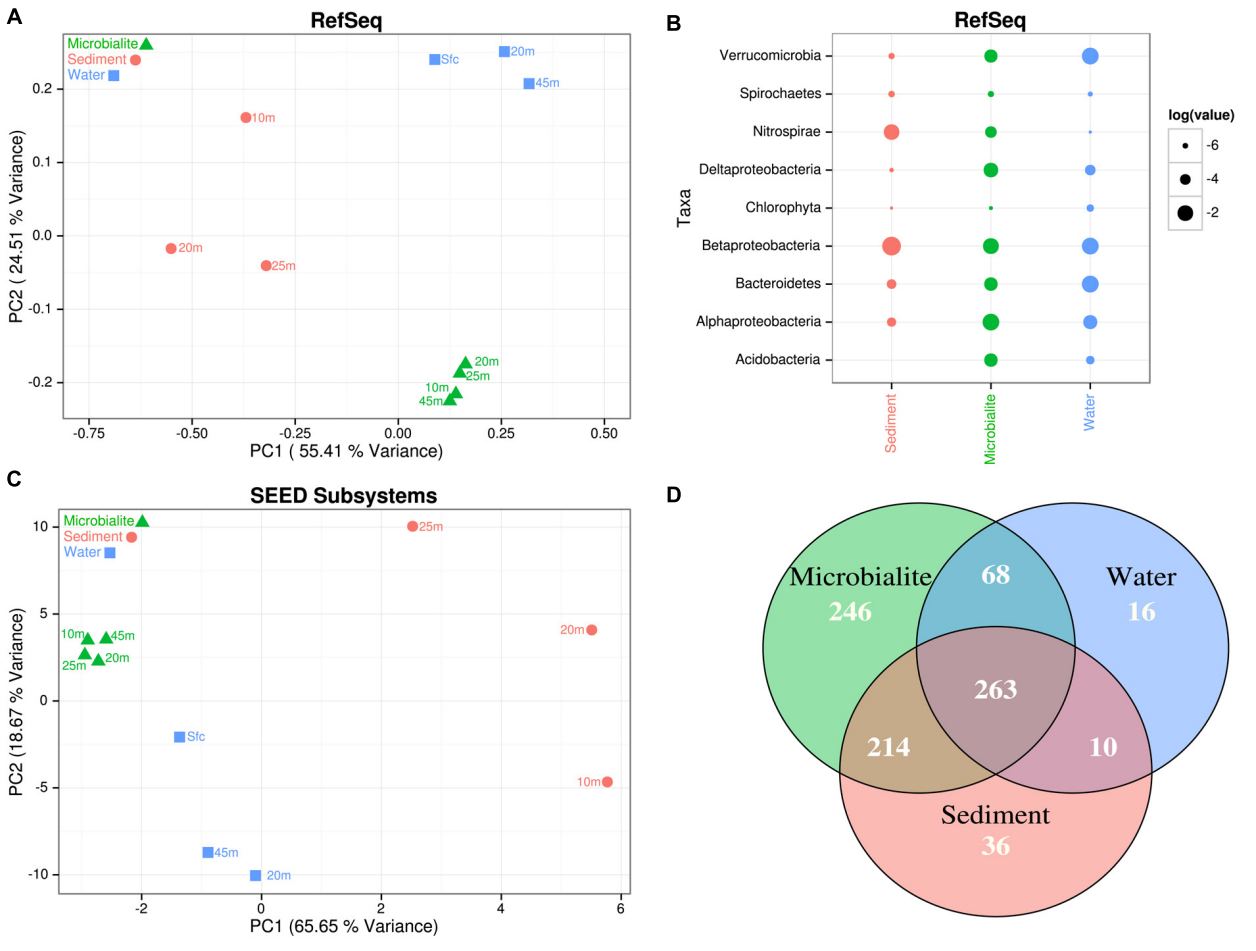
**Pavilion Lake microbial community composition and metabolic potential relative to the adjacent sediments and water.** **(A)** PCA plot of the normalized RefSeq classifications. Clustering was done using a ward matrix and Bray-Curtis distance cut-offs, bootstrapped with one thousand replicates. **(B)** Dotplot of the normalized RefSeq class-level in log relative abundances. **(C)** PCA plot of the normalized SEED Subsystems (level II classifications). Clustering was done using a ward matrix and Bray-Curtis distance cut-offs, bootstrapped with one thousand replicates. **(D)** Venn diagram of the MetaCyc pathway abundances for all the microbialite, water and sediment metagenomes normalized by pathway gene abundances and pathway size.

The microbial community structure and metabolic potential of Pavilion Lake microbialites were statistically different from those in the surrounding environment (e.g., water and sediment metagenomes), based on principle component analyses of RefSeq taxonomic classifications (**Figure 2A**), SEED (**Figure 2C**), and MetaCyc functional gene assignments. STAMP ANOVA of the RefSeq classifications identified thirteen bacterial classes that were significantly enriched in microbialites over the surrounding environment (**Table 2**, *p* < 0.01). ANOVA using STAMP on the highest level classification (level I) in the SEED database indicates that membrane transport, aromatic metabolism, motility, potassium metabolism, cell signaling and virulence genes are significantly enriched in microbialites over the surrounding environment (**Table 3**, *p* < 0.05). MetaCyc functional gene assignments suggest many shared pathways (263) among samples from the water (filters), microbialites and sediments with 246 pathways distinct to Pavilion microbialites (**Figure 2D**). These observations support the idea that microbialite associated microbes are distinct and are not being seeded or introduced (at least not recently), from surrounding environments.

**Table 2:** Taxonomic classes (RefSeq) that are overrepresented in microbialites relative to the surrounding environment by ANOVA using STAMP.

Bacteria	*p*-value (corr)	Effect size	Fil: Avg Rfreq (%)	Filr: SD (%)	MB: Avg Rfreq. (%)	MB: SD (%)	Sed: Avg Rfreq. (%)	Sed: SD (%)
*Acidobacteriia*	1.96E-03	0.889	0.314	0.164	0.839	0.139	0	0
*Acidobacteria (Unclassified)*	8.34E-05	0.972	0.096	0.053	0.849	0.094	0	0
*Alphaproteobacteria*	4.52E-04	0.947	7.708	0.266	19.768	2.196	2.679	2.008
*Chloroflexi (class)*	9.30E-05	0.973	0.449	0.142	1.481	0.116	0	0
*Deferribacteres (class)*	1.13E-03	0.917	0.059	0.014	0.117	0.02	0	0
*Deinococci*	1.82E-03	0.892	0.335	0.131	0.613	0.081	0	0
*Deltaproteobacteria*	1.81E-04	0.961	2.151	0.933	7.85	0.448	0.398	0.563
*Gemmatimonadetes*	1.39E-03	0.904	0.208	0.16	0.797	0.114	0	0
*Gloeobacteria*	7.68E-05	0.972	0.056	0.038	0.36	0.03	0	0
*Ktedonobacteria*	1.49E-03	0.9	0.076	0.042	0.31	0.062	0	0
*Solibacteres*	1.16E-04	0.973	0.386	0.279	3.346	0.331	0	0
*Thermomicrobia (class)*	1.44E-03	0.905	0.058	0.03	0.481	0.111	0	0
*Thermotogae (class)*	5.03E-03	0.845	0.044	0.023	0.138	0.006	0.023	0.033


*STAMP ANOVA tested significant differences between multiple groups (microbialite, sediments, and filters) using a post-hoc test (Tukey–Kramer at 0.95), an effect size (Eta-squared), a p-value (<0.01) and a multiple-test correction of Benjamini–Hochberg FDR. Results are for RefSeq classifications that significantly differed (*p* < 0.01) for microbialite over the surrounding environments (sediments and filters). Avg Rfreq, Average relative frequency; SD, Standard deviation; p-val corr, p-value ANOVA corrected; Unclass, Unclassified; MB, Microbialite; Sed, Sediment.*

*Avg Rfreq, Average relative frequency; SD, Standard deviation. P-val corr, p-value ANOVA corrected (By Benjamini–Hochberg FDR); Fil, Filter represents surrounding water; MB, Microbialite; Sed, Sediment.*

**Table 3:** Functional annotations (SEED subsystem level I) that are overrepresented in microbialites relative to the surrounding environment by ANOVA using STAMP.

Seed subsystem	*p*-val (corr)	Effect Size	Fil: Avg Rfeq (%)	Fil: SD (%)	MB: Avg Rfeq (%)	MB: SD (%)	Sed: Avg Rfeq (%)	Sed: SD (%)
Membrane transport	8.12E-03	0.793	1.998	0.157	2.514	0.112	0.941	0.577
Metabolism of aromatics	3.00E-04	0.934	1.27	0.074	1.775	0.111	0.872	0.109
Motility and chemotaxis	3.60E-03	0.649	0.606	0.025	1.063	0.039	0.454	0.356
Potassium metabolism	6.40E-03	0.81	0.182	0.035	0.296	0.013	0.084	0.068
Regulation and cell signaling	1.82E-03	0.874	1.09	0.028	1.506	0.079	0.978	0.133
Virulence, disease, and defense	3.45E-04	0.928	2.069	0.195	2.997	0.057	1.167	0.327


*STAMP ANOVA tested significant differences between multiple groups (microbialite, sediments, and filters) using a *post hoc* test (Tukey–Kramer at 0.95), an effect size (Eta-squared), a p-value (<0.01) and a multiple-test correction of Benjamini–Hochberg FDR. Results are for RefSeq classifications that significantly differed (*p* < 0.01) for microbialite over the surrounding environments (sediments and filters). Avg Rfreq, Average relative frequency; SD, Standard deviation; p-val corr, p-value ANOVA corrected; MB, Microbialite; Sed, Sediment.*

*Avg Rfreq, Average relative frequency; SD, Standard deviation.*

*p-val corr, p-value ANOVA corrected (Benjamini-Hochberg FDR).*

*Fil, Filter represents surrounding water; MB, Microbialite. Sed: Sediment.*

The microbial communities and metabolic potential of the microbialites differed between the sediment and water samples. Compared to the microbialites, the sediment metagenomic data had more sequences assigned to the taxonomic groups *Nitrospirae*, *Betaproteobacteria* and *Spirochaetia*; whereas, metagenomic data from the water had more sequences assigned to *Betaproteobacteria, Bacterioidetes, Verrucomicrobia*, and phototrophic eukaryotes (e.g., *Chlorophyceae*; **Figure 2B**). Although the depth of sequences was not the same across microbialites, sediments and water, it was adequate to clearly show that the microbialite community was distinct from those in the surrounding environments. These findings are consistent with previous works that demonstrate fundamental differences in microbial taxa between microbialite-associated communities and others. Russell et al. (2014) found taxonomically distinct microbial communities in non-lithifying soft-mat biofilms and microbialites in Pavilion Lake. As well, metagenomic analysis of marine microbialites in Highbourne Cay showed distinctly different communities associated with lithifying and non-lithifying microbial mats (Khodadad and Foster, 2012)

### Core Microbialite Microbial Community Structure and Metabolic Potential

**FIGURE 3:**
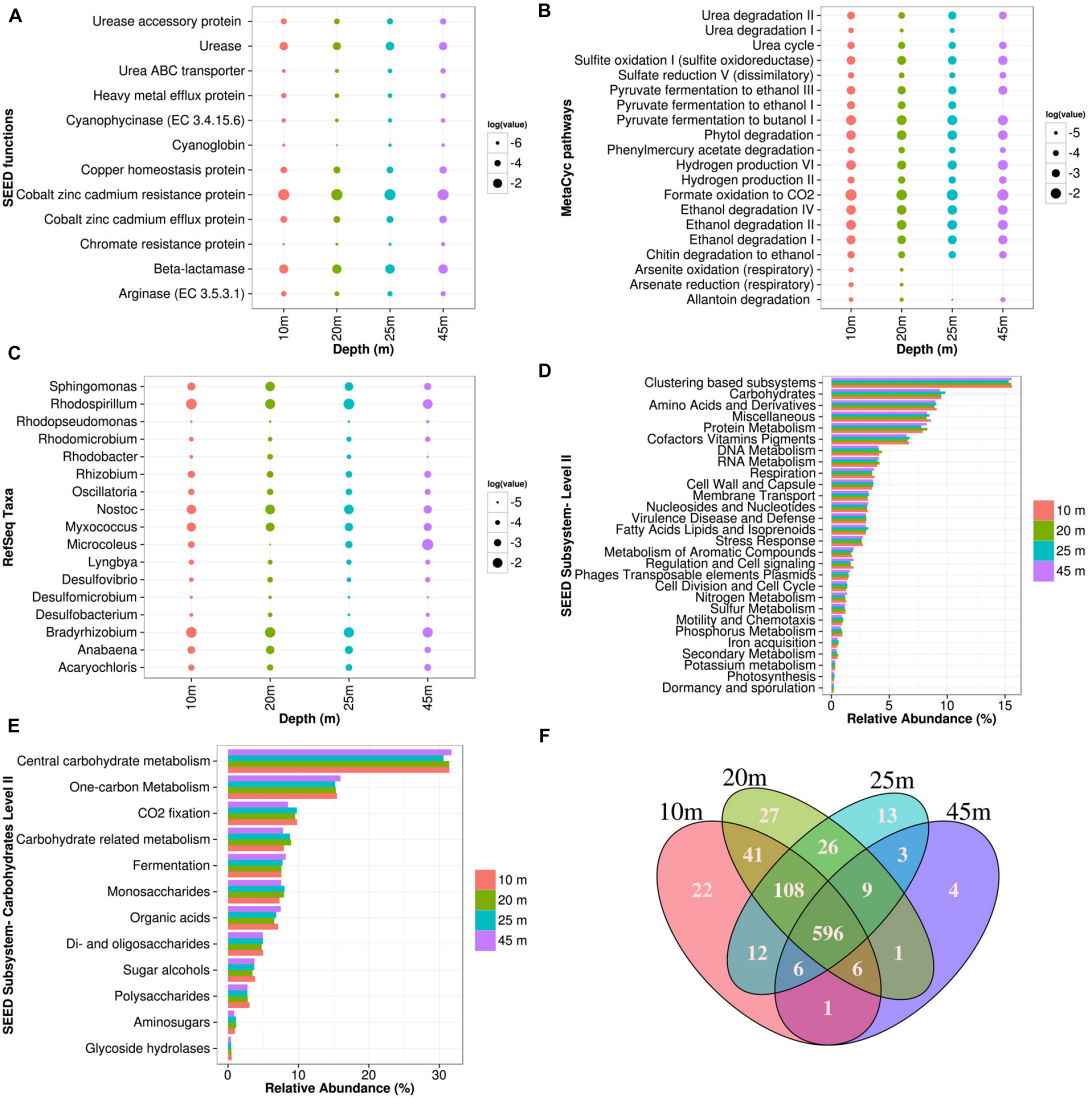
**Pavilion Lake microbial community composition and metabolic potential across microbialite morphologies.** Microbialite metagenomes are listed as a function of depth in meters. **(A)** Dotplot of the normalized SEED subsystem functions relating to urea metabolism (e.g., urease, ABC transport), heavy metal detoxification (e.g., efflux, resistance), antibiotic resistance (e.g beta-lactamases), and cyanobacteria related functions (e.g., cyanoglobin, cyanophycinase, copper homeostasis) in log relative abundances. **(B)** Dotplot of the normalized MetaCyc pathways relating to urea metabolism, sulfite oxidation, dissimilatory sulfate reduction, heavy metal detoxification (e.g., arsenite/arsenate oxidation/reduction, phenylmercury acetate metabolism), hydrogen production, primary alcohol fermentation/degradation in log relative abundances. **(C)** Dotplot of the normalized RefSeq classifications relating to *Alphaproteobacteria, Deltaproteobacteria*, and filamentous cyanobacterial genera. **(D)** Microbialite metagenome functional annotation ranking using SEED subsystem (level I). **(E)** Microbialite metagenome carbohydrate related SEED subsystem (level II) functional annotations. **(F)** Venn diagram of the MetaCyc pathway abundances for all the microbialite metagenomes normalized by pathway gene abundances and pathway size.

Microbialite morphology in Pavilion Lake changes predictably with depth, however the metabolic potential and microbial community remains similar. PCA of RefSeq (**Figure 2A**) and SEED (**Figure 2C**) classifications indicate that the microbial community and metabolic potential of microbialite metagenomes cluster closely together, regardless of morphology or depth (**Figure 3D**). Across morphologies, >80% of the MetaCyc pathways predicted by the microbialite metagenomes are shared (596, **Figure 3F**), with few (<30) distinct pathways within a Pavilion Lake microbialite morphotype.

Based on RefSeq taxonomic classification ANOVA using STAMP, the microbialites of Pavilion Lake were dominated by sequences assigned to *Proteobacteria* and *Acidobacteria* (**Figure 2B**). For example, sequences assigned to the classes *Alphaproteobacteria*, *Deltaproteobacteria*, *Acidobacteriia*, and *Gloeobacteria* were significantly more abundant in microbialite metagenomes than in the water or sediment metagenomes (**Table 2**; *p* < 0.05). The dominance of sequences associated with members of the phyla *Proteobacteria* (mainly *Alphaproteobacteria* and *Deltaproteobacteria* classes, **Figure 2B**) is consistent with results from other marine and freshwater microbialite communities (Havemann and Foster, 2008; Breitbart et al., 2009; Goh et al., 2009; Khodadad and Foster, 2012; Mobberley et al., 2013), suggesting that despite geographical and environmental differences, microbialite microbial communities have similar members suggesting a globally shared microbial community structure.

The microbialites had significantly more sequences classified as *Alphaproteobacteria*, both in photoheterotrophic and heterotrophic functional groups, relative to sequences in the neighboring environments (e.g., water and sediment metagenomes) (**Table 2**; *p* < 0.05; **Figure 2B**). Microbialite alphaproteobacterial specific contigs were assigned to genera of photoheterotrophic (e.g., *Rhodobacter*, *Rhodomicrobium, Rhodopseudomonas*, and *Rhodospirillum*), heterotrophic (e.g., *Sphingomonas*) and nitrogen-fixing (e.g., *Bradyrhizobium*, and *Rhizobium*) bacteria (**Figure 3C**). Alphaproteobacterial based nitrogen fixation complements cyanobacterial nitrogen fixation in microbialites likely because of cyanobacterial diel cycles (Havemann and Foster, 2008). *Alphaproteobacteria* were also found to be the dominant OTU phylotype in Pavilion Lake microbialites in prior pyrosequencing and clone-library amplicon studies (Chan et al., 2014; Russell et al., 2014), consistent with our data on protein-coding gene abundances in the microbialite metagenomes.

Deltaproteobacterial associated sequences within the microbialite metagenomes were assigned to genera of dissimilatory sulfate reducing (e.g., *Desulfobacterium* and *Desulfovibrio*) and heterotrophic (e.g., *Myxococcus*) bacteria (**Figure 3C**). MetaCyc dissimilatory sulfate pathways were abundant across the different microbialite morphologies in Pavilion Lake (**Figure 3B**). Sulfate-reducing deltaproteobacteria are often found where carbonates precipitate, and are important drivers of the “alkalinity engine,” by pushing the saturation index via increasing alkalinity (Gallagher et al., 2012). Hydrogen production and formate oxidation to carbon dioxide are predicted by the microbialite metagenomes (**Figure 3B**). Potential electron donors for sulfate reducing deltaproteobacteria in Pavilion Lake microbialites include acetate, lactate, hydrogen, and formate. Whether sulfate reduction helps or hinders carbonate precipitation depends on the electron donor; hydrogen and formate likely promote precipitation, whereas, other organic carbon sources likely lead to dissolution (Gallagher et al., 2012). Future stable isotope probing studies could reveal which compounds are used as electron donors by the sulfate-reducers. *Myxococcus* spp. are abundant in a variety of microbialite-forming systems and can directly precipitate carbonate through the release of ammonium, which can increase alkalinity favoring carbonate precipitation (Ben Chekroun et al., 2004; Jimenez-Lopez et al., 2011). Analysis of the Pavilion Lake microbialite metagenomes supports prior metagenomic and amplicon investigations of microbialites that show members of the *Deltaproteobacteria* include dissimilatory sulfate-reducers (Havemann and Foster, 2008; Breitbart et al., 2009; Goh et al., 2009; Khodadad and Foster, 2012; Mobberley et al., 2013; Wong et al., 2015).

Sequences associated with filamentous cyanobacterial mat-builders from the genera *Anabaena, Lyngbya, Microcoleus, Nostoc, Oscillatoria* and the planktonic *Cyanothece* and *Acrayochoris* were found in all microbialite morphologies (**Figure 3C**). Pathways for synthesis of cyanoglobin and cyanophycin, as well as copper metabolism, were associated with cyanobacterial mat-builders in all microbialites (**Figure 3B**). Cyanoglobin is a peripheral membrane protein that binds oxygen with high affinity, is highly expressed under low oxygen and could be restricted to some strains of *Nostoc* sp. and *Anabaena* sp. (Hill et al., 1996). Cyanophycin is formed in filamentous cyanobacteria in response to low or changing DIC to O_2_ ratios (Liang et al., 2014). Copper homeostasis genes were abundant in microbialites, which is common for cyanobacterial derived mats, as copper is essential for growth (Varin et al., 2012) but also toxic at levels ≥10 mM (Burnat et al., 2009). The microbialite metagenome indicates that the metabolic potential of filamentous cyanobacterial mats is adaptive to metal homeostasis (e.g., copper), as well as carbon and oxygen limitation (e.g., cyanoglobin and cyanophycin).

### Novel Metabolic Potential Within Microbialites

Urealytic metabolism has been hypothesized to be involved in microbialite formation due to its carbonate precipitating effects, but its detection in microbialites has remained elusive (Castanier et al., 1999). MetaCyc and SEED subsystems indicate that urea ABC transporters, arginase and ureases are found in similar abundances across Pavilion Lake microbialites (**Figures 3A,B**), implying the presence of urea metabolism which may be playing a role in precipitation. *Gamma* and *Deltaproteobacteria* specific urease beta subunits and urease accessory proteins (UreD/F) have only been identified in the Pavilion Lake microbialite metagenomes. The linkage of urease related genes to *Proteobacteria* was unexpected due to the strong experimental evidence that *Firmicutes* (mainly *Bacillus* sp.) are the dominant taxa contributing urease related genes (Boquet et al., 1973; Hammes et al., 2003; Lee, 2003; Dick et al., 2006; Dhami et al., 2013).

Antibiotic and heavy-metal resistance pathways associated with *Proteobacteria* were found within the microbialites based on RefSeq classification (**Figures 3A,B**). These included antibiotic resistance pathways such as beta-lactamases (class A) that were assigned to the *Alpha*, *Beta*, and *Gamma* classes of *Proteobacteria* (**Figure 2A**). Genes related to antibiotic resistance could be in response to toxic organic molecules produced by cyanobacterial mats (Neilan et al., 2013). SEED functions and MetaCyc pathways related to heavy-metal detoxification were abundant in microbialites (**Figure 3A**). The occurrence of cobalt–zinc–cadmium resistance proteins, efflux pump proteins, phenylmercury acetate degradation, and chromate resistance was similar across morphologies while arsenite oxidation and arsenate reduction pathways were not found at depths deeper than 25 m (**Figure 3B**). Heavy-metal resistance contigs were taxonomically assigned to *Alpha*, *Beta* and *Gamma* classes of *Proteobacteria*. Pavilion Lake has low levels of zinc (0.01–0.03 mg L^-1^) and undetectable levels of cobalt, iron, arsenic and cadmium (Lim et al., 2009). Heavy-metal resistance genes may be involved in resistance, homeostasis or sequestration of metals. Antibiotic resistance has also been linked to heavy-metal stress, suggesting that resistance to one can lead to resistance to the other in complex bacterial communities (Nisanian et al., 2014).

Recently published Shark Bay microbialite metagenomes suggest high prevalence of genes associated with heavy-metal resistance including genes for arsenic metabolism (e.g., reductase and resistance genes; Ruvindy et al., 2015). Arsenite oxidation and arsenate reduction genes were also found amongst MetaCyc pathways in only the 10 to 20 m microbialites in Pavilion Lake (**Figure 3B**). Arsenic cycling has been suggested to be a prominent feature in ancient microbial mats over 2.7 billion years old (Sforna et al., 2014). Our data from Pavilion Lake microbialites suggest that heavy-metal resistance could be a general feature of microbialites globally, which may also provide cross-protection against antibiotics (Nisanian et al., 2014).

The metabolic potential of Pavilion Lake microbialites predict primary alcohol fermentation pathways (e.g., butanol and ethanol biosynthesis) (**Figure 3B**) based on genes that are taxonomically assigned to *Alpha-* and *Beta-proteobacteria*. Pyruvate, phytol, and chitin fermentation appear to be the main predicted pathways for the generation of primary alcohols (e.g., ethanol, butanol). Primary alcohol fermentation has been linked to microbialite dissolution; however, fermentation also provides substrates that fuel dissimilatory sulfate reduction, which can precipitate carbonate (Dupraz and Visscher, 2005; Gallagher et al., 2012) and which could offset carbonate lost by fermentation. Further stable-isotope experiments are needed to confirm the metabolic potential of primary alcohol fermentation predicted by the microbialite metagenomes. Although not previously recognized, members of the *Proteobacteria* appear to be major constituents of Pavilion Lake microbialites, potentially providing important metabolic roles, such as resistance to antibiotics and heavy-metals, and primary alcohol fermentation. Further investigation into the nature of their role in microbialite formation is warranted.

### Photosynthetic and Heterotrophic Metabolic Potential Associated with Carbonate Precipitation in Pavilion Lake

**FIGURE 4:**
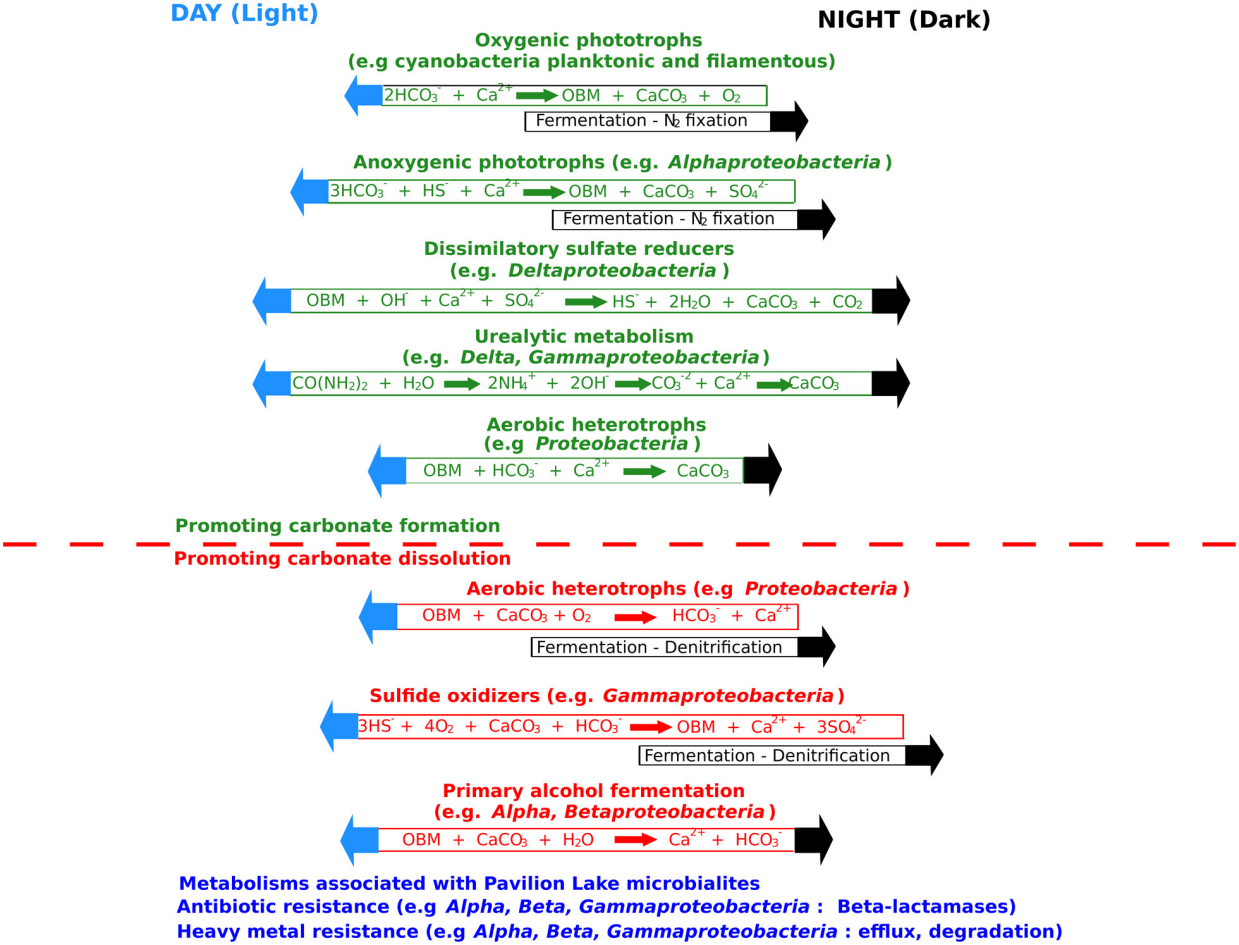
**A summary of the factors affecting carbonate precipitation and dissolution as inferred from the Pavilion Lake microbialite metabolic potential and community composition.** OBM is organic biomass. Adapted from Dupraz et al. (2009).

The metabolic potential of Pavilion Lake microbialites appears to be dominated by heterotrophy relative to phototrophy. Sequences related to photosynthesis, including those encoding photosystems and electron transport proteins, were ranked 28th out of 29th possible SEED subsystems (**Figure 3D**). In contrast, pathways related to carbohydrate metabolism (carbon-related pathways) were ranked second and accounted for ∼9% of the contigs. Among the carbon-related pathways, ∼45% were related to central (TCA cycle) and one-carbon metabolism (e.g., serine–glyoxlate cycle), while another ∼45% were related to degradation (e.g., fermentation, glycoside hydrolases, and other hydrolytic enzymes; **Figure 3E**). Only ∼10% of the microbialite-specific contigs were annotated as carbon-fixation related (e.g., Calvin–Benson cycle) (**Figure 3E**). Stable-isotope studies suggest that photosynthetic processes are linked to carbonate precipitation in shallow (<25 m) microbialites (Brady et al., 2009; Omelon et al., 2013), even though the metabolic potential is dominated by heterotrophic processes. However, Omelon et al. (2013) and Theisen et al. (2015) also suggested that heterotrophs contribute to the lifthification of microbialites in Pavilion Lake by triggering additional carbonate precipitation. Microbialite formation relating to carbonate precipitation in Pavilion Lake is associated with cyanobacterial photosynthesis with contribution from heterotrophic processes such as urealytic metabolism, dissimilatory sulfate reduction and heterotrophic mat degradation (i.e., EPS related carbonate inhibition; **Figure 4**; Dupraz et al., 2009).

### Viral Community and Viral Defense

**FIGURE 5:**
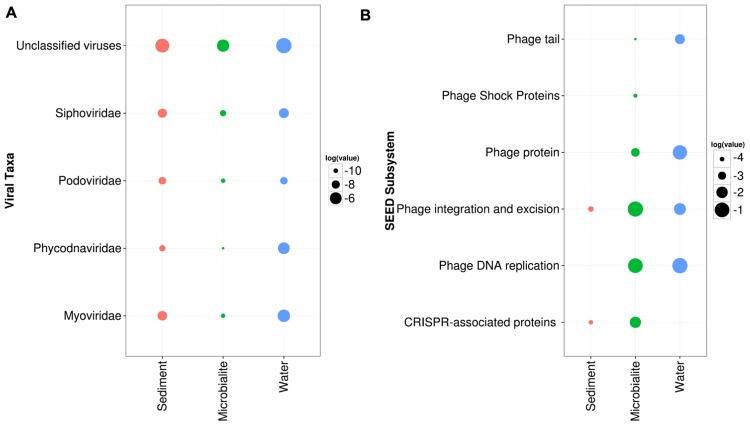
**Pavilion Lake viral community composition and metabolic potential.** **(A)** Dotplot of normalized RefSeq viral taxonomic groups in relation to the surrounding environments (e.g., sediments and water) in log relative abundances. **(B)** Dotplot of the normalized SEED subsystem in relation to the surrounding environments (e.g., sediments and water) in log relative abundances.

In the cellular fraction from the water, viral sequences represented >1% of reads; whereas, in total DNA extracted from microbialites, viral sequences comprised >0.05% of reads. ANOVA in STAMP based on RefSeq classification confirmed that virus sequences were relatively more abundant in the water than in microbialites or sediments (**Figure 5A**). Specifically, T4-like phage (e.g., *Myoviridae*) and large algal viruses (e.g., *Phycodnaviridae*) dominated the viral sequences in the water and were more abundant than in the microbialites and sediments (**Figure 5A**). The low proportion of viral reads in the microbialite data may be biased by the lack of dsDNA viral genomes in the RefSeq database from microbialites compared to water. Viruses in the water appeared to have higher abundances of proteins related to phage structure (tail fibers), phage replication and phage DNA replication (**Figure 5B**).

An active role for phages in the microbialites is suggested by the higher relative abundances of predicted genes involved with CRISPRs, phage shock and phage excision (**Figure 5B**). CRISPR *cas* genes were associated with the following taxonomic groups: *Chloroflexi* (e.g., *Dehalococcoides*), *Deltaproteobacteria* (e.g., *Myxococcus* and *Desulfuromonadales*), filamentous cyanobacteria (e.g., *Anabaena, Nostoc, Rivularia*) and *Firmicutes* (e.g., *Clostridia*) based on tBLASTx (1e^-3^) analysis. Likewise, more putative genes involved in phage integration and excision occurred in the microbialites than in the nearby environment (**Figure 5B**). Also, CRISPRs were predicted to be associated with key members involved in microbialite formation, such as filamentous cyanobacteria and *Myxococcus* sp., implying that the microbialite community is under continuous selective pressure from viral infection.

It is important to emphasize that the viral DNA was from the cellular fraction (between 0.2 and 120 μm) captured on filters, suggesting that most viral sequences were from infected cells or from viruses attached to particles. It is not uncommon for filters with pore sizes much larger than viruses to contain many viral sequences (Zeigler Allen et al., 2012). The most abundant viral contigs in the water were for T4-like cyanophages and phycodnaviruses (**Figure 5A**). Although gene-specific primers targeted to these groups (Chen and Suttle, 1995; Filée et al., 2005) failed to amplify DNA, it would suggest that the viruses were evolutionarily distinct from the viruses these primers target.

Consistent with reports for other microbialites (Desnues et al., 2008), relatively few viral sequences were recovered in this study. Yet, the occurrence of phage integration and CRISPR-*cas* sequences implies that the Pavilion Lake microbialites are under selection from viral infection. It is likely that the relative abundance of viral sequences has been underestimated because of the lack of representative viral sequences from microbialites in databases.

## Conclusion

This study demonstrates that the microbial community profile and metabolic potential of modern freshwater microbialites in Pavilion Lake are distinct from those in neighboring sediments and water, consistent with previous findings of spatial variation in microbialite systems. These results confirm the notion that the microbialite communities are not being continuously seeded by organisms from the surrounding environment. Our data further suggests a unique microbialite microbial community that encodes a functional guild which is distinctive and likely related to its overall function of carbonate precipitation.

Differences among these metagenomes can be attributed to differing selection pressures among environments, with the microbialite community comprised of taxa essential for microbialite growth, as well as opportunists taking advantage of nutrients and the matrix supplied by filamentous cyanobacterial mats. These findings are consistent with photosynthetic influences on carbonate precipitation by filamentous cyanobacteria, with likely contributions by proteobacteria and acidobacteria.

Pavilion Lake microbialites are enriched for pathways that include heavy-metal and antibiotic resistance, urealytic metabolism as well as primary alcohol fermentation. These pathways are associated with members of the *Proteobacteria*, which are numerically dominant and likely convey resistance to toxins and heavy metals, and may influence carbonate formation through photosynthesis and urea metabolism. This hypothesis is consistent with previous suggestions of heterotrophic contributions to lithification of Pavilion Lake microbialites. Evidence for urealytic metabolism identified here may suggest an important role for this metabolism in carbonate precipitation (Castanier et al., 1999), which has not been reported previously in microbialites.

The prevalence of CRISPR-*cas* systems and phage excision genes imply that the microbialites are under selective pressure from viral infection. In particular, the presence of CRISPRs assigned to taxa that precipitate carbonates (*Cyanobacteria*, *Deltaproteobacteria* and *Firmicutes*) suggest that viruses play an important previously unknown role in the microbialite communities in Pavilion Lake.

## Conflict of Interest Statement

The authors declare that the research was conducted in the absence of any commercial or financial relationships that could be construed as a potential conflict of interest.

## Footnotes

1 http://broadinstitute.github.io/picard

2 http://www.theseed.org

3 http://www.ncbi.nlm.nih.gov/refseq/

4 http://metacyc.org/

5 http://metagenomics.anl.gov/

## References

[B1] Allen M. A. Goh F. Burns B. P. Neilan B. A. (2009). Bacterial, archaeal and eukaryotic diversity of smooth and pustular microbial mat communities in the hypersaline lagoon of Shark Bay. *Geobiology* 7 82–96. 10.1111/j.1472-4669.2008.00187.x 19200148

[B2] Aury J. M. Cruaud C. Barbe V. Rogier O. Mangenot S. Samson G. (2008). High quality draft sequences for prokaryotic genomes using a mix of new sequencing technologies. *BMC Genomics* 9:603 10.1186/1471-2164-9-603 PMC2625371 19087275

[B3] Ben Chekroun K. Rodriguez-Navarro C. Gonzalez-Muñoz M. T. Arias J. M. Cultrone G. Rodriguez-Gallego M. (2004). Precipitation and growth morphology of calcium carbonate induced by *Myxococcus xanthus*: implications for regognition of bacterial carbonates. *J. Sedimentary Res.* 74 868–876. 10.1306/050504740868

[B4] Bentley D. R. Balasubramanian S. Swerdlow H. P. Smith G. P. Milton J. Brown C. G. (2008). Accurate whole human genome sequencing using reversible terminator chemistry. *Nature* 6 53–59. 10.1038/nature07517 18987734 PMC2581791

[B5] Boisvert S. Laviolette F. Corbeil J. (2010). Ray: simultaneous assembly of reads from a mix of high-throughput sequencing technologies. *J. Comput. Biol.* 11 1519–1533. 10.1089/cmb.2009.0238 20958248 PMC3119603

[B6] Boisvert S. Raymond F. Godzaridis E. Laviolette F. Corbeil J. (2012). Ray Meta: scalable de novo metagenome assembly and profiling. *Genome Biol.* 13:R122 10.1186/gb-2012-13-12-r122 PMC4056372 23259615

[B7] Boquet E. Boronat A. Ramos-Cormenzana A. (1973). Production of calcite (calcium carbonate) crystals by soil bacteria is a general phenomenon. *Nature* 246 527–529. 10.1038/246527a0

[B8] Bosak T. Liang B. Wu T. D. Templer S. P. Evans A. Vali H. (2012). Cyanobacterial diversity and activity in modern conical microbialites. *Geobiology* 10 384–401. 10.1111/j.1472-4669.2012.00334.x 22713108

[B9] Brady A. L. Laval B. Lim D. S. S. Slater G. F. (2014). Autotrophic and heterotrophic associated biosignatures in modern freshwater microbialites over seasonal and spatial gradients. *Org. Geochem.* 67 8–18. 10.1016/j.orggeochem.2013.11.013

[B10] Brady A. L. Slater G. Laval B. Lim D. S. (2009). Constraining carbon sources and growth rates of freshwater microbialites in Pavilion Lake using 14C analysis. *Geobiology* 7 544–555. 19702837 10.1111/j.1472-4669.2009.00215.x

[B11] Brady A. L. Slater G. F. Omelon C. R. Southam G. Druschel G. Andersen D. T. (2010). Photosynthetic isotope biosignatures in laminated micro-stromatolitic and non-laminated nodules associated with modern, freshwater microbialites in Pavilion Lake, B.C. *Chem. Geol.* 274 56–67. 10.1016/j.chemgeo.2010.03.016

[B12] Breitbart M. Hoare A. Nitti A. Siefert J. Haynes M. Dinsdale E. (2009). Metagenomic and stable isotopic analyses of modern freshwater microbialites in Cuatro Cienegas, Mexico. *Environ. Microbiol.* 11 16–34. 10.1111/j.1462-2920.2008.01725.x 18764874

[B13] Burnat M. Diestra E. Esteve I. Solé A. (2009). In situ determination of the effects of lead and copper on cyanobacterial populations in microcosms. *PLoS ONE* 4:e6204 10.1371/journal.pone.0006204 PMC2703825 19593432

[B14] Burne R. V. Moore L. S. (1987). Microbialites: organosedimentary deposits of benthic microbial communities. *Palaios* 2 241–254. 10.2307/3514674

[B15] Burns B. P. Goh F. Allen M. Neilan B. A. (2004). Microbial diversity of extant stromatolites in the hypersaline marine environment of Shark Bay, Australia. *Environ. Microbiol.* 6 1096–1101. 10.1111/j.1462-2920.2004.00651.x 15344935

[B16] Cantarel B. L. Erickson A. R. VerBerkmoes N. C. Erickson B. K. Carey P. A. (2011). Strategies for metagenomic-guided whole-community proteomics of complex microbial environments. *PLoS ONE* 6:e27173 10.1371/journal.pone.0027173 PMC3223167 22132090

[B17] Castanier S. Le Metayer-Levrel G. Perthuisot J. P. (1999). Ca-carbonates precipitation and limestone genesis - the microbiogeologist point of view. *Sedim. Geol.* 126 9–23.

[B18] Centeno C. M. Legendre P. Beltran Y. Alcantara-Hernandez R. J. Lidstrom U. E. Ashby M. N. (2012). Microbialite genetic diversity and composition related to environmental variables. *FEMS Microbiol. Ecol.* 82 724–735. 10.1111/j.1574-6941.2012.01447.x 22775797

[B19] Chan O. W. Bugler-Lacap D. C. Biddle J. F. Lim D. S. S. McKay C. P. Pointing S. B. (2014). Phylogenetic diversity of a microbialite reef in a cold alkaline freshwater lake. *Can. J. Microbiol.* 6 391–398. 10.1139/cjm-2014-0024 24861562

[B20] Chen F. Suttle C. A. (1995). Amplification of DNA polymerase gene fragments from viruses infecting microalgae. *Appl. Environ. Microbiol.* 61 1274–1278. 7747950 10.1128/aem.61.4.1274-1278.1995 PMC167383

[B21] Couradeau E. Benzerara K. Moreira D. Gérard E. Kaźmierczak J. Tavera R. (2011). Prokaryotic and eukaryotic community structure in field and cultured microbialites from the alkaline Lake Alchichica (Mexico). *PLoS ONE* 6:e28767 10.1371/journal.pone.0028767 PMC3237500 22194908

[B22] Desnues C. Rodriguez-Brito B. Rayhawk S. Kelley S. Tran T. Haynes M. (2008). Biodiversity and biogeography of phages in modern stromatolites and thrombolites. *Nature* 452 340–343. 10.1038/nature06735 18311127

[B23] Dhami N. K. Reddy M. S. Mukherjee A. (2013). Biomineralization of calcium carbonate polymorphs by the bacterial strains isolated from calcareous sites. *J. Microbiol. Biotechnol.* 23 707–714. 10.4014/jmb.1212.11087 23648862

[B24] Dick J. De Windt W. De Graef B. Saveyn H. Van der Meeren P. De Belie N. (2006). Bio-deposition of a calcium carbonate layer on degraded limestone by *Bacillus species*. *Biodegradation* 17 357–367. 10.1007/s10532-005-9006-x 16491305

[B25] Dupraz C. Reid R. P. Braissant O. Decho A. W. Norman R. S. Visscher P. T. (2009). Processes of carbonate precipitation in modern microbial mats. *Earth Sci. Rev.* 96 141–162. 10.1016/j.earscirev.2008.10.005

[B26] Dupraz C. Visscher P. T. (2005). Microbial lithification in marine stromatolites and hypersaline mats. *Trends Microbiol.* 13 429–438. 10.1016/j.tim.2005.07.008 16087339

[B27] Ferris F. G. Thompson J. B. Beveridge T. J. (1997). Modern freshwater microbialites from Kelly Lake, British Columbia, Canada. *Palaios* 12 213–219. 10.2307/3515423

[B28] Filée J. Tétart F. Suttle C. A. Krisch H. M. (2005). Marine T4-type bacteriophages, a ubiquitous component of the dark matter of the biosphere. *Proc. Natl. Acad. Sci. U.S.A.* 102 12471–12476. 10.1073/pnas.0503404102 16116082 PMC1194919

[B29] Gallagher K. L. Kading T. J. Braissant O. Dupraz C. Visscher P. T. (2012). Inside the alkalinity engine: the role of electron donors in the organomineralization potential of sulfate-reducing bacteria. *Geobiology* 10 518–530. 10.1111/j.1472-4669.2012.00342.x 22925453

[B30] Gischler E. Gibson M. A. Oschmann W. (2008). Giant holocene freshwater microbialites, laguna bacalar, quintana roo, Mexico. *Sedimentology* 55 1293–1309. 10.1111/j.1365-3091.2007.00946.x

[B31] Goh F. Allen M. A. Leuko S. Kawaguchi T. Decho A. W. Burns B. P. (2009). Determining the specific microbial populations and their spatial distribution within the stromatolite ecosystem of Shark Bay. *ISME J.* 3 383–396. 10.1038/ismej.2008.114 19092864

[B32] Grotzinger J. P. Knoll A. H. (1999). Stromatolites in Precambrian carbonates: evolutionary mileposts or environmental dipsticks? *Annu. Rev. Earth Planet Sci.* 27 313–358. 10.1146/annurev.earth.27.1.313 11543060

[B33] Hammes F. Boon N. de Villiers J. Verstraete W. Siciliano S. D. (2003). Strain-specific ureolytic microbial calcium carbonate precipitation. *Appl. Environ. Microbiol.* 69 4901–4909. 10.1128/AEM.69.8.4901-4909.2003 12902285 PMC169139

[B34] Havemann S. A. Foster J. S. (2008). Comparative characterization of the microbial diversities of an artificial microbialite model and a natural stromatolite. *Appl. Environ. Microbiol.* 74 7410–7421. 10.1128/AEM.01710-08 18836014 PMC2592906

[B35] Hill D. R. Belbin T. J. Thorsteinsson M. V. Bassam D. Brass S. Ernst A. (1996). GlbN (cyanoglobin) is a peripheral membrane protein that is restricted to certain *Nostoc* spp. *J. Bacteriol* 178 6587–6598. 8932316 10.1128/jb.178.22.6587-6598.1996 PMC178546

[B36] Jimenez-Lopez C. Chekroun K. B. Jroundi F. Rodríguez-Gallego M. Arias J. M. González-Muñoz M. T. (2011). *Myxococcus xanthus* colony calcification: an study to better understand the processes involved in the formation of this stromatolite-like structure. *Adv. Strom. Geobiol.* 131 161–181. 10.1007/978-3-642-10415-2_11

[B37] Khodadad C. L. Foster J. S. (2012). Metagenomic and metabolic profiling of nonlithifying and lithifying stromatolitic mats of Highborne Cay, The Bahamas. *PLoS ONE* 7:e38229 10.1371/journal.pone.0038229 PMC3360630 22662280

[B38] Kiełbasa S. M. Wan R. Sato K. Horton P. Frith M. C. (2011). Adaptive seeds tame genomic sequence comparison. *Genome Res.* 3 487–493. 10.1101/gr.113985.110 21209072 PMC3044862

[B39] Konwar K. M. Hanson N. W. Pagé A. P. Hallam S. J. (2013). MetaPathways: a modular pipeline for constructing pathway/genome databases from environmental sequence information. *BMC Bioinform.* 14:202 10.1186/1471-2105-14-202 PMC3695837 23800136

[B40] Langmead B. Salzberg S. L. (2012). Fast gapped-read alignment with Bowtie 2. *Nat. Methods* 9 357–359. 10.1038/nmeth.1923 22388286 PMC3322381

[B41] Laval B. Cady S. L. Pollack J. C. McKay C. P. Bird J. S. Grotzinger J. P. (2000). Modern freshwater microbialite analogues for ancient dendritic reef structures. *Nature* 407 626–629. 10.1038/35036579 11034210

[B42] Lee Y. N. (2003). Calcite production by *Bacillus amyloliquefaciens* CMB01. *J. Microbiol.* 41 345–348.

[B43] Liang B. Wu T. D. Sun H. J. Vali H. Guerquin-Kern J. L. Wang C. H. (2014). Cyanophycin mediates the accumulation and storage of fixed carbon in non-heterocystous filamentous cyanobacteria from coniform mats. *PLoS ONE* 9:e88142 10.1371/journal.pone.0088142 PMC3917874 24516596

[B44] Lim D. S. S. Brady A. L. Pavilion Lake Research Project (Plrp) Team Abercromby A. F. Andersen D. T. Andersen M. (2011). A historical overview of the Pavilion Lake Research Project - Analog science and exploration in an underwater environment. *Geol. Soc. Am. Special Papers* 483 85–115. 10.1130/2011.2483(07)

[B45] Lim D. S. S. Laval B. E. Slater G. Antoniades D. Forrest A. L. Pike W. (2009). Limnology of Pavilion Lake, B.C., Canada - Characterization of a microbialite forming environment. *Fundam. Appl. Limnol.* 173 329–351. 10.1111/gbi.12121

[B46] López-García P. Kazmierczak J. Benzerara K. Kempe S. Guyot F. Moreira D. (2005). Bacterial diversity and carbonate precipitation in the giant microbialites from the highly alkaline Lake Van, Turkey. *Extremophiles* 9 263–274. 10.1007/s00792-005-0457-0 15959626

[B47] Lundberg J. McFarlane D. A. (2011). Subaerial freshwater phosphatic stromatolites in Deer Cave, Sarawak—A unique geobiological cave formation. *Geomorphology* 128 57–72. 10.1016/j.geomorph.2010.12.022

[B48] Maliva G. R. Missimer M. T. Leo C. K. Statom A. R. Dupraz C. Lynn M. (2000). Unusual calcite stromatolites and pisoids from a landfill leachate collection system. *Geology* 28 931–934. 10.1130/0091-7613(2000)28<931:UCSAPF>2.0.CO;2

[B49] Merz M. U. E. (1992). The biology of carbonate precipitation by cyanobacteria. *Facies* 26 81–101. 10.1007/BF02539795

[B50] Meyer F. D. Paarmann M. D’Souza R. Olson E. M. Glass M. Kubal T. (2008). The Metagenomics RAST server - A public resource for the automatic phylogenetic and functional analysis of metagenomes. *BMC Bioinform.* 9:386 10.1186/1471-2105-9-386 PMC2563014 18803844

[B51] Mobberley J. M. Khodadad C. L. Foster J. S. (2013). Metabolic potential of lithifying cyanobacteria-dominated thrombolitic mats. *Photosynth. Res.* 118 125–140. 10.1007/s11120-013-9890-6 23868401 PMC5766932

[B52] Neilan B. A. Pearson L. A. Muenchhoff J. Moffitt M. C. Dittmann E. (2013). Environmental conditions that influence toxin biosynthesis in cyanobacteria. *Environ. Microbiol.* 5 1239–1253. 10.1111/j.1462-2920.2012.02729.x 22429476

[B53] Nisanian M. Holladay S. D. Karpuzoglu E. Kerr R. P. Williams S. M. Stabler L. (2014). Exposure of juvenile Leghorn chickens to lead acetate enhances antibiotic resistance in enteric bacterial flora. *Poult. Sci.* 93 891–897. 10.3382/ps.2013-03600 24706966

[B54] Omelon C. R. Brady A. L. Slater G. F. Laval B. Lim D. S. S. Southam G. (2013). Microstructure variability in freshwater microbialites, Pavilion Lake, Canada. *Palaeogeogr. Palaeoclimatol. Palaeoecol.* 392 62–70. 10.1016/j.palaeo.2013.08.017

[B55] Parks D. H. Beiko R. G. (2010). Identifying biologically relevant differences between metagenomic communities. *Bioinformatics* 26 715–721. 10.1093/bioinformatics/btq041 20130030

[B56] Perry R. S. Mcloughlin N. Lynne B. Y. Sephton M. A. Oliver J. D. Perry C. C. (2007). Defining biominerals and organominerals: direct and indirect indicators of life. *Sedim. Geol.* 201 157–179. 10.1016/j.sedgeo.2007.05.014

[B57] Power I. M. Wilson S. A. Dipple G. M. Southam G. (2011). Modern carbonate microbialites from an asbestos open pit pond, Yukon, Canada. *Geobiology* 9 180–195. 10.1111/j.1472-4669.2010.00265.x 21231993

[B58] R Development Core Team (2015). *R: A Language and Environment for Statistical Computing*. Vienna: The R Foundation for Statistical Computing.

[B59] Reid R. P. Visscher P. T. Decho A. W. Stolz J. F. Bebout B. M. Dupraz C. (2000). The role of microbes in accretion, lamination and early lithification of modern marine stromatolites. *Nature* 406 989–999. 10.1038/35023158 10984051

[B60] Russell J. A. Brady A. L. Cardman Z. Slater G. F. Lim D. S. S. Biddle J. F. (2014). Prokaryote populations of extant microbialites along a depth gradient in Pavilion Lake, British Columbia, Canada. *Geobiology* 12 250–264. 10.1111/gbi.12082 24636451

[B61] Ruvindy R. White R. A. III Neilan B. A. Burns B. P. (2015). Unravelling core microbial metabolisms in the hypersaline microbial mats of Shark Bay using high-throughput metagenomics. *ISME J Adv.* 29:2015 10.1038/ismej.2015.87 PMC4681862 26023869

[B62] Santos F. Peña A. Nogales B. Soria-Soria E. del Cura M. A. González-Martín J. A. (2010). Bacterial diversity in dry modern freshwater stromatolites from Ruidera Pools Natural Park. *Spain. Syst. Appl. Microbiol.* 33 209–221. 10.1016/j.syapm.2010.02.0 20409657

[B63] Schmieder R. Edwards R. (2011). Quality control and preprocessing of metagenomic datasets. *Bioinformatics* 27 863–864. 10.1093/bioinformatics/btr026 21278185 PMC3051327

[B64] Schmieder R. Lim Y. W. Rohwer F. Edwards R. (2010). TagCleaner: identification and removal of tag sequences from genomic and metagenomic datasets. *BMC Bioinform.* 11:341 10.1186/1471-2105-11-341 PMC2910026 20573248

[B65] Schopf J. W. (2006). Fossil evidence of Archean life. *Philos. Trans. R. Soc. Lond. B. Biol. Sci. B* 361 869–885. 10.1098/rstb.2006.1834 16754604 PMC1578735

[B66] Sforna M. C. Philippot P. Somogyi A. van Zuilen M. A. Medjoubi K. Schoepp-Cothenet B. (2014). Evidence for arsenic metabolism and cycling by microorganisms 2.7 *billion years ago*. *Nat. Geosci.* 7 811–815. 10.1038/ngeo2276

[B67] Suttle C. A. (2005). Viruses in the Sea. *Nature* 437 356–361. 10.1038/nature04160 16163346

[B68] Suttle C. A. Chan A. M. Cottrell M. T. (1991). Use of ultrafiltration to isolate viruses from seawater which are pathogens of marine phytoplankton. *Appl. Environ. Microbiol.* 57 721–726. 16348439 10.1128/aem.57.3.721-726.1991 PMC182786

[B69] Suzuki R. Shimodaira H. (2006). Pvclust: an R package for assessing the uncertainty in hierarchical clustering. *Bioinformatics* 22 1540–1542. 10.1093/bioinformatics/btl117 16595560

[B70] Theisen C. H. Sumner D. Y. Mackey T. J. Lim D. S. Brady A. L. Slater G. F. (2015). Carbonate fabrics in the modern microbialites of Pavilion Lake: two suites of microfabrics that reflect variation in microbial community morphology, growth habit, and lithification. *Geobiology* 4 357–372. 10.1111/gbi.12134 25809931

[B71] Untergasser A. (2008). *DNA Miniprep using Ctab Untergasser’s Lab*. Available at: http://www.untergasser.de/lab/protocols/miniprep_dna_ctab_v1_0.htm [Accessed July 10 2010].

[B72] Varin T. Lovejoy C. Jungblut A. D. Vincent W. F. Corbeil J. (2012). Metagenomic analysis of stress genes in microbial mat communities from Antarctica and the high Arctic. *Appl. Environ. Microbiol.* 78 549–559. 10.1128/AEM.06354-11 22081564 PMC3255749

[B73] White R. A. III Blainey P. C. Fan H. C. Quake S. R. (2009). Digital PCR provides sensitive and absolute calibration for high throughput sequencing. *BMC Genomics* 10:116 10.1186/1471-2164-10-116 PMC2667538 19298667

[B74] White R. A. III Grassa C. J. Suttle C. A. (2013a). First draft genome sequence from a member of the genus *Agrococcus*, isolated from modern microbialites. *Genome Announc.* 1:e391–e413 10.1128/genomeA.00391-13 PMC3695436 23814108

[B75] White R. A. III Grassa C. J. Suttle C. A. (2013b). Draft Genome Sequence of *Exiguobacterium pavilionensis* strain RW-2, with wide thermal, salinity, and pH tolerance, isolated from modern freshwater microbialites. *Genome Announc.* 1:e597–e613 10.1128/genomeA.00597-513 PMC3738901 23929485

[B76] White R. A. III Power I. A. Dipple G. M. Southern G. Suttle C. A. (2015). Metagenomic analysis reveals that modern microbialites and polar microbial mats have similar taxonomic and functional potential. *Front. Microbiol.* 6:966 10.3389/fmicb.2015.00966 PMC4585152 26441900

[B77] White R. A. III Suttle C. A. (2013). The Draft Genome Sequence of Sphingomonas paucimobilis Strain HER1398 (*Proteobacteria*), Host to the Giant PAU Phage, Indicates That It Is a Member of the Genus Sphingobacterium (*Bacteroidetes*). *Genome Announc.* 1:e598–e613 10.1128/genomeA.00598-13 PMC3738902 23929486

[B78] Wickham H. (2009). *ggplot2: Elegant Graphics for Data Analysis*. New York, NY: Springer.

[B79] Wong H. L. Smith D. L. Visscher P. T. Burns B. P. (2015). Niche differentiation of bacterial communities at a millimeter scale in Shark Bay microbial mats. *Sci. Rep.* 5:15607 10.1038/srep15607 PMC4620479 26499760

[B80] Zeigler Allen L. Allen E. E. Badger J. H. McCrow J. P. Paulsen I. T. Elbourne L. D. (2012). Influence of nutrients and currents on the genomic composition of microbes across an upwelling mosaic. *ISME J.* 6 1403–1414. 10.1038/ismej.2011.201 22278668 PMC3379637

