# Supplementary material for: Metagenomic Analysis Suggests Modern Freshwater Microbialites Harbor a Distinct Core Microbial Community
Source: Front Microbiol. 2016 Jan 28;6:1531. doi: 10.3389/fmicb.2015.01531 (PMC4729913; doi:10.3389/fmicb.2015.01531)
Supplement: Supplementary file 1 [file Data_Sheet_1.PDF]

Modern freshwater microbialites reveals distinct microbial community structure and metabolic potential that differs from the surrounding environment

Richard Allen White III[1*], Amy M Chan[2], Allyson Brady[5], Greg F Slater[5], Darlene S. Lim[6] and Curtis A. Suttle[1,2,3,4]

Department of Microbiology & Immunology, University of British Columbia, Vancouver, BC Canada[1]
Department of Earth, Ocean & Atmospheric Sciences, University of British Columbia, Vancouver, BC Canada[2]
Department of Botany, University of British Columbia, Vancouver, BC Canada[3]
Canadian Institute for Advanced Research[4]
School of Geography & Earth Sciences, McMaster University, Hamilton, Ontario, Canada[5]
SETI Institute, Mountain View, CA, USA[6]

*Currently at the Pacific Northwest National Laboratory (PNNL), Richland, WA USA

Address correspondence to Curtis A. Suttle, suttle@science.ubc.ca

Supplemental Material

Supplemental Tables and figures

Table S1

Pavilion Lake metagenome assembly statistics.

	Microbialites				Water			Sediments*		
**Depth**	**10 m**	**20 m**	**25 m**	**45 m**	**Sfc****	**20 m**	**25 m**	**10 m**	**20 m**	**25 m**
# Contigs or Reads	510,415	881,449	499,134	179,215	43,909	19,977	32,086	120,680	544,185	94,150
Total Length (Bp)	115,196,707	178,186,326	111,159,198	41,483,863	8,537,385	3,836,213	5,930,888	12,560,841	61,059,259	10,421,166
N50 >500bp	647	752	717	668	649	734	608	(-)	(-)	(-)
Avg >500bp	721	783	760	736	776	753	644	(-)	(-)	(-)
Med >500bp	571	633	616	621	524	670	575	(-)	(-)	(-)
Largest (Bp)	3882	6079	2965	1964	7125	4003	1880	190	190	190
G+C%	60.8	57.9	60.1	60.3	47	52.8	52.6	59.6	57.7	52.2

*Not assembled reads only

**Sfc: surface water filter (1 to 10 m) (-) not available.

Table S2

Taxonomic classes (RefSeq) that are overrepresented in microbialites relative to the surrounding environment by ANOVA using STAMP.

Bacteria	p-val (corr)	Effect size	Water: Avg Rfreq (%)	Water: SD (%)	MB: Avg Rfreq. (%)	MB: SD (%)	Sed:Avg Rfreq. (%)	Sed: SD (%)
Acidobacteriia	1.96E-003	0.889	0.314	0.164	0.839	0.139	0.000	0.000
Acidobacteria (Unclass)	8.34E-005	0.972	0.096	0.053	0.849	0.094	0.000	0.000
Alphaproteobacteria	4.52E-004	0.947	7.078	0.266	19.768	2.196	2.679	2.008
Chloroflexi (class)	9.30E-005	0.973	0.449	0.142	1.481	0.116	0.000	0.000
Deferribacteres (class)	1.13E-003	0.917	0.059	0.014	0.117	0.020	0.000	0.000
Deinococci	1.82E-003	0.892	0.335	0.131	0.613	0.081	0.000	0.000
Deltaproteobacteria	1.81E-004	0.961	2.151	0.933	7.850	0.448	0.398	0.563
Gemmatimonadetes	1.39E-003	0.904	0.208	0.160	0.797	0.114	0.000	0.000
Gloeobacteria	7.68E-005	0.972	0.056	0.038	0.360	0.030	0.000	0.000
Ktedonobacteria	1.49E-003	0.900	0.076	0.042	0.310	0.062	0.000	0.000
Solibacteres	1.16E-004	0.973	0.386	0.279	3.346	0.331	0.000	0.000
Thermomicrobia (class)	1.44E-003	0.905	0.058	0.030	0.481	0.111	0.000	0.000
Thermotogae (class)	5.03E-003	0.845	0.044	0.023	0.138	0.006	0.023	0.033
**Archaea**	**p-val (corr)**	**Effect size**	**Water: Avg Rfreq (%)**	**Water: SD (%)**	**MB: Avg Rfreq. (%)**	**MB: SD (%)**	**Sed: Avg Rfreq. (%)**	**Sed: SD (%)**
Euryarchaeota (unclass)	8.67E-004	0.928	0.010	0.007	0.039	0.004	0.000	0.000
Halobacteria	8.11E-004	0.931	0.043	0.017	0.172	0.029	0.000	0.000
Korarchaeota (Unclass)	4.49E-004	0.945	0.000	0.000	0.009	0.002	0.000	0.000
Methanomicrobia	4.79E-004	0.943	0.062	0.036	0.312	0.044	0.000	0.000
Methanopyri	3.85E-003	0.860	0.000	0.000	0.005	0.002	0.000	0.000
Thermococci	2.64E-003	0.877	0.014	0.013	0.051	0.007	0.000	0.000
Thermoplasmata	3.70E-003	0.863	0.002	0.003	0.012	0.003	0.000	0.000

Avg Rfreq: Average relative frequency. SD: Standard Deviation. p-val corr: p-value ANOVA corrected. Unclass: Unclassified. MB: Microbialite. Sed: Sediment.

Table S3

Functional annotations (SEED subsystem level I) that are overrepresented in microbialites relative to the surrounding environment by ANOVA using STAMP

	P-val (corr)	Effect Size	Water:Avg Rfeq (%)	Water: SD (%)	MB:Avg Rfeq (%)	MB: SD (%)	Sed:Avg Rfeq (%)	Sed: SD (%)
Membrane Transport	8.12E-003	0.793	1.998	0.157	2.514	0.112	0.941	0.577
Metabolism of Aromatics	3.00E-004	0.934	1.270	0.074	1.775	0.111	0.872	0.109
Motility and Chemotaxis	3.60E-002	0.649	0.606	0.025	1.063	0.039	0.454	0.356
Potassium metabolism	6.40E-003	0.810	0.182	0.035	0.296	0.013	0.084	0.068
Regulation and Cell signaling	1.82E-003	0.874	1.090	0.028	1.506	0.079	0.978	0.133
Virulence, Disease and Defense	3.45E-004	0.928	2.069	0.195	2.997	0.057	1.167	0.327

Avg Rfreq: Average relative frequency. SD: Standard Deviation. p-val corr: p-value ANOVA corrected.
MB: Microbialite. Sed: Sediment.

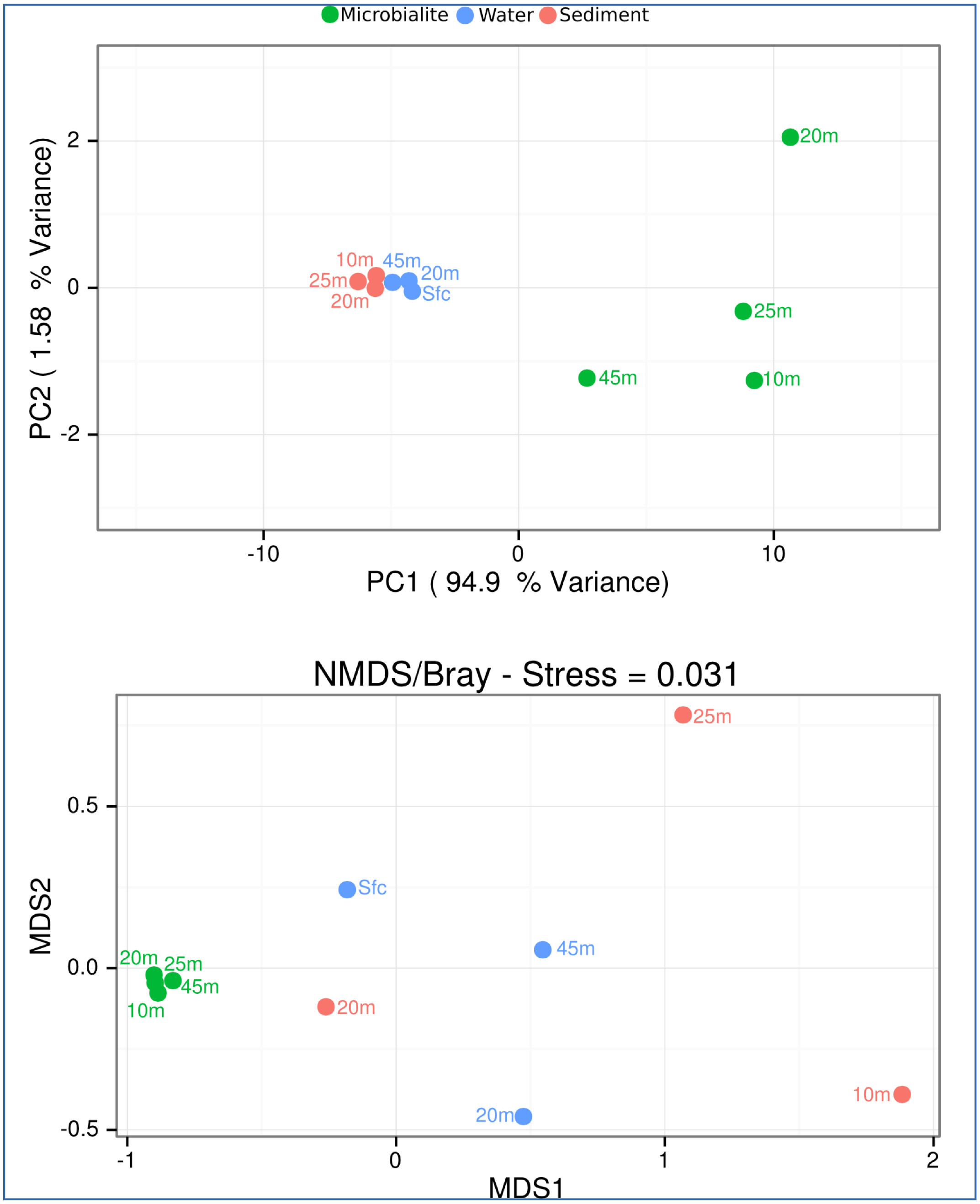

Figure S1

MetaCyc pathway annotations for Pavilion Lake metagenomes.

(Top) PCA by ward clustering followed by bootstrapping of thousand replicates using Bray-Curtis distance metric.

(Bottom) NMDS by ward clustering followed by bootstrapping of thousand replicates using Bray-Curtis distance metric.

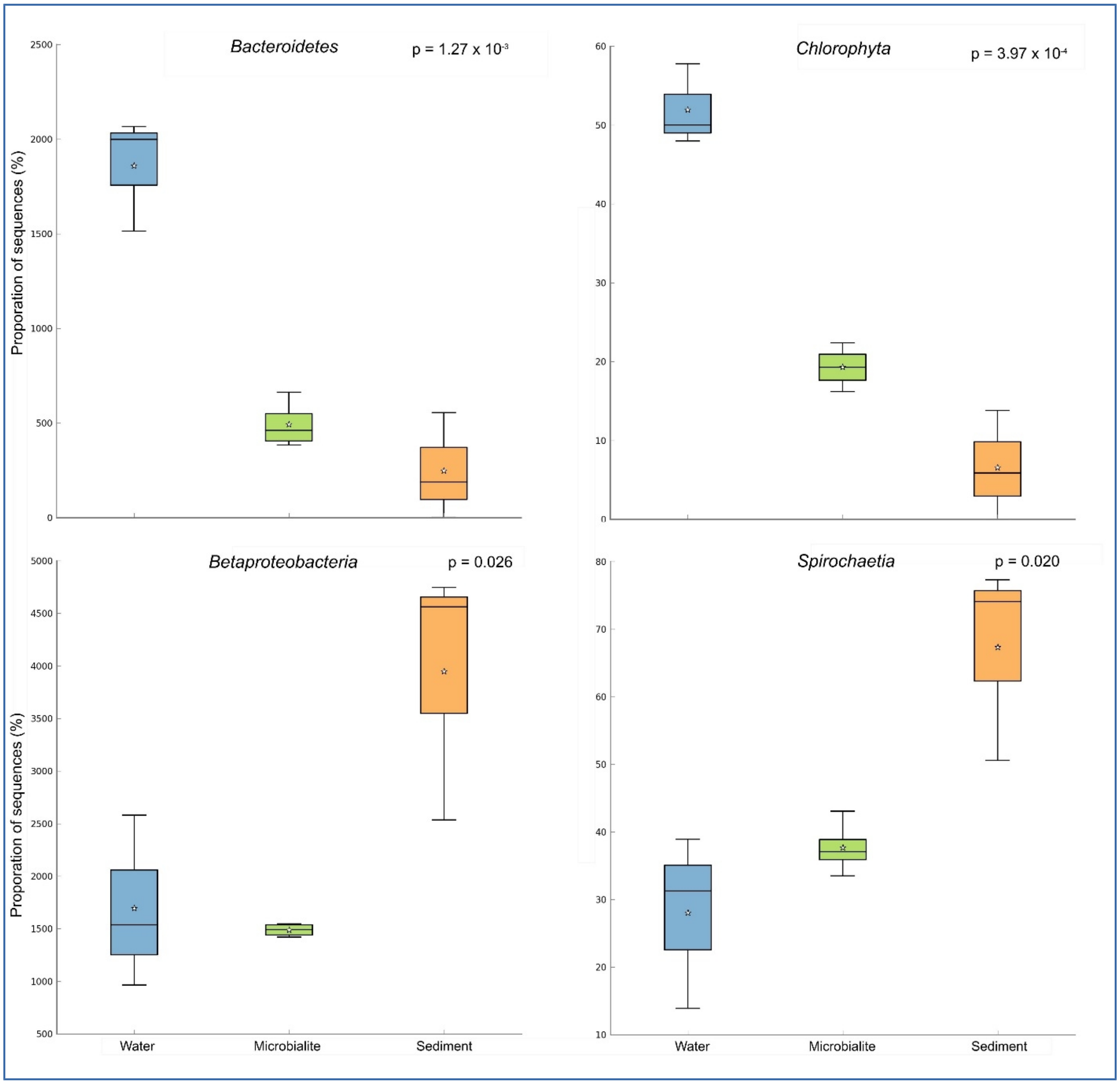

Figure S2

Box and whisker plots for significant taxa represented in water filters and sediments by RefSeq classification using ANOVA in STAMP based on multiple groups.

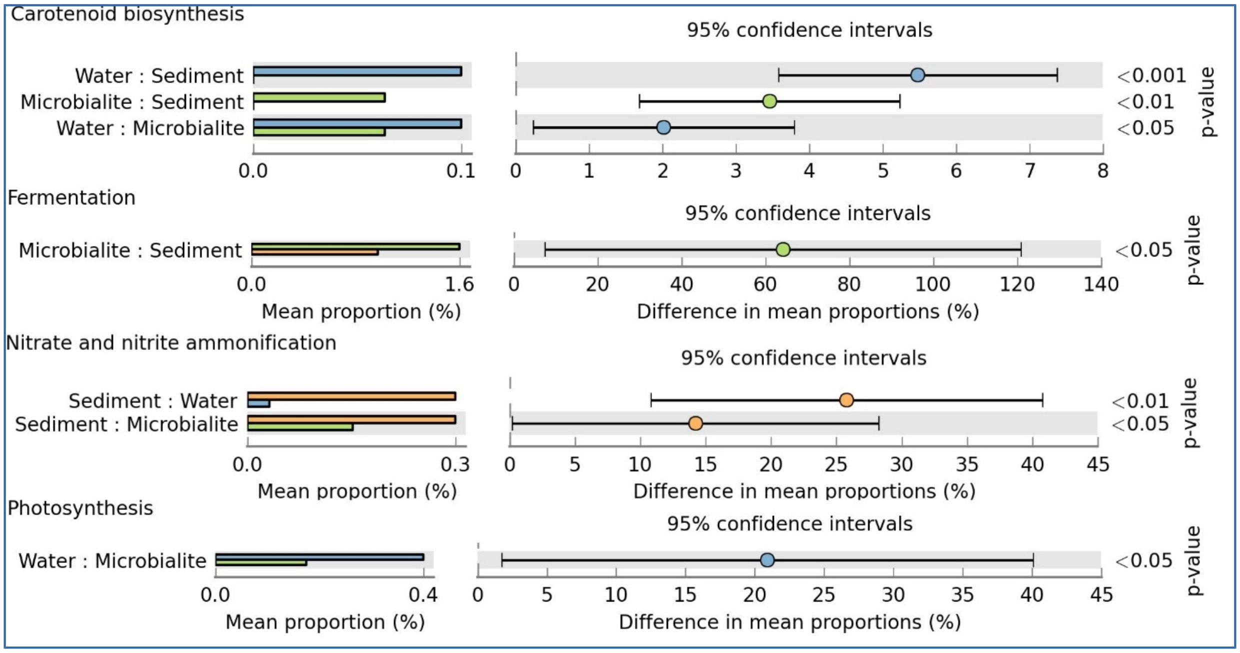

Figure S3

Post-havoc confidence interval plots functional classification for represented in surrounding environment (water filters and sediments).

SEED subsystems classifications are based on ANOVA in STAMP based on multiple groups. Microbialites are labeled in green, followed by sediments in orange and water in blue.

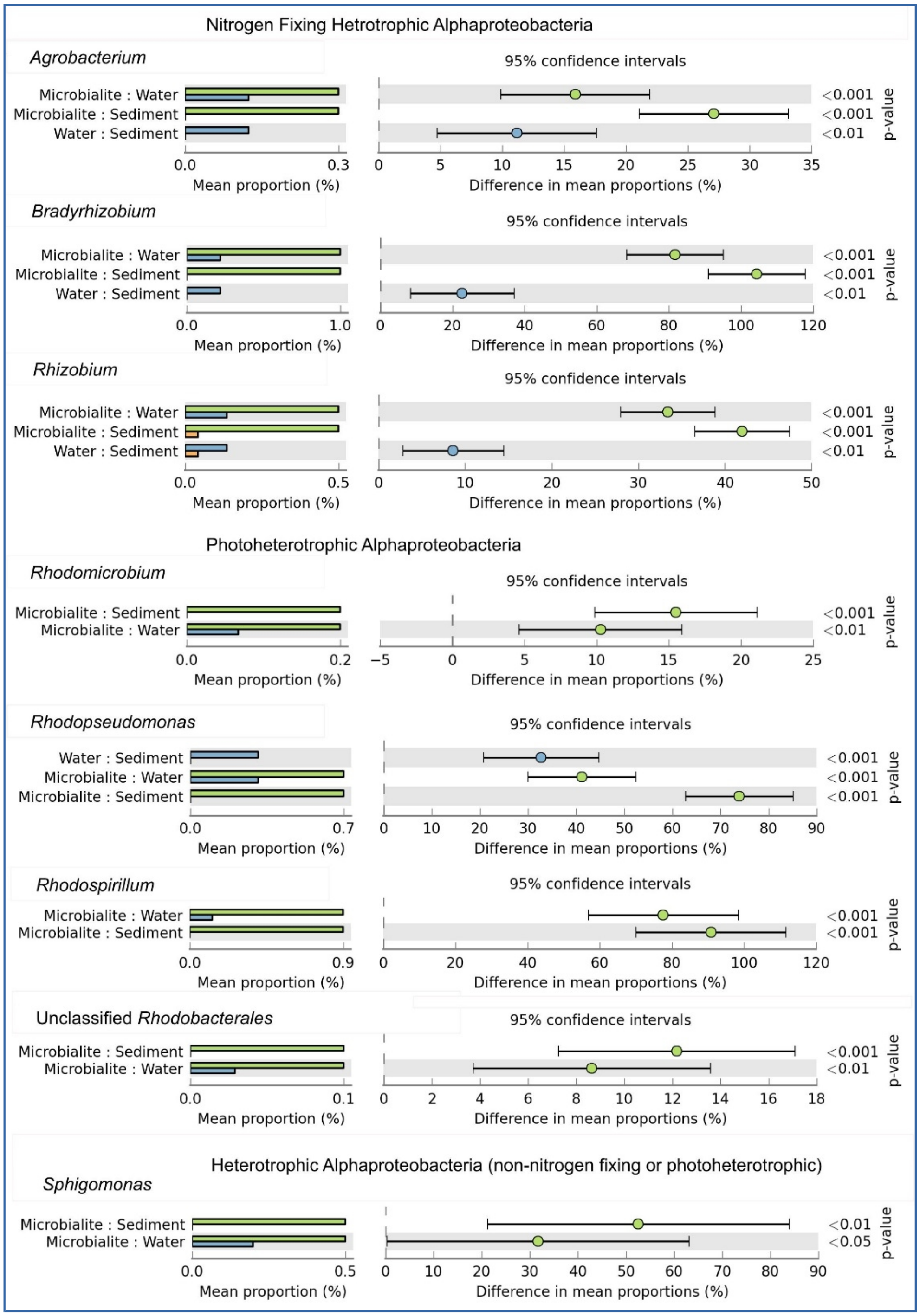

Figure S4

Post-havoc confidence interval plots for *Alphaproteobacteria* using RefSeq classification. Based on ANOVA in STAMP for significant genus-level members using multiple groups. Microbialites are labeled in green, followed by sediments in orange and water in blue.

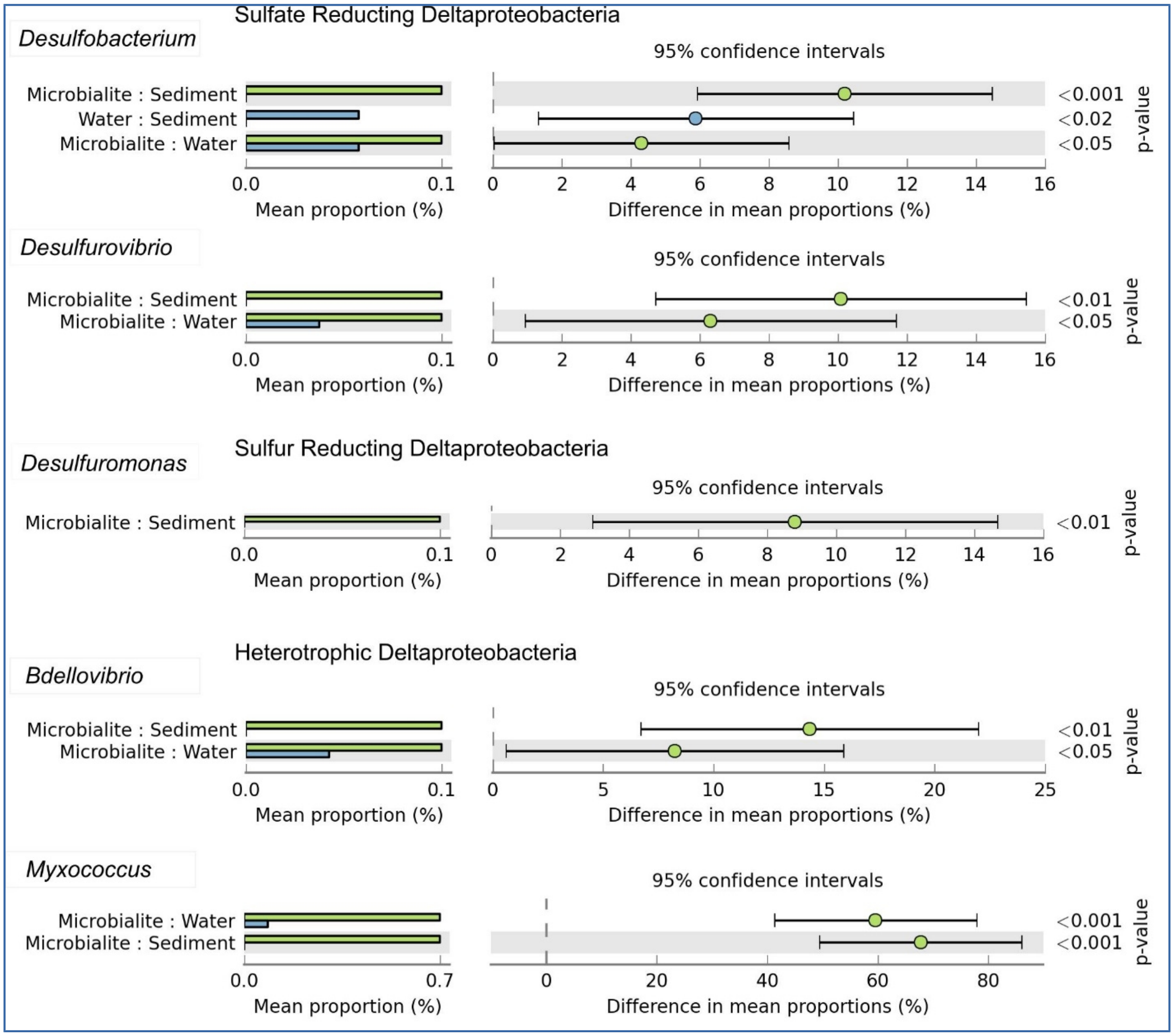

Figure S5

Post-havoc confidence interval plots for *Deltaproteobacteria* using RefSeq classification. Based on ANOVA in STAMP for significant genus-level members represented in Pavilion Lake metagenomes (microbialite, water filter and sediment) using multiple groups. Microbialites are labeled in green, followed by sediments in orange and water in blue.

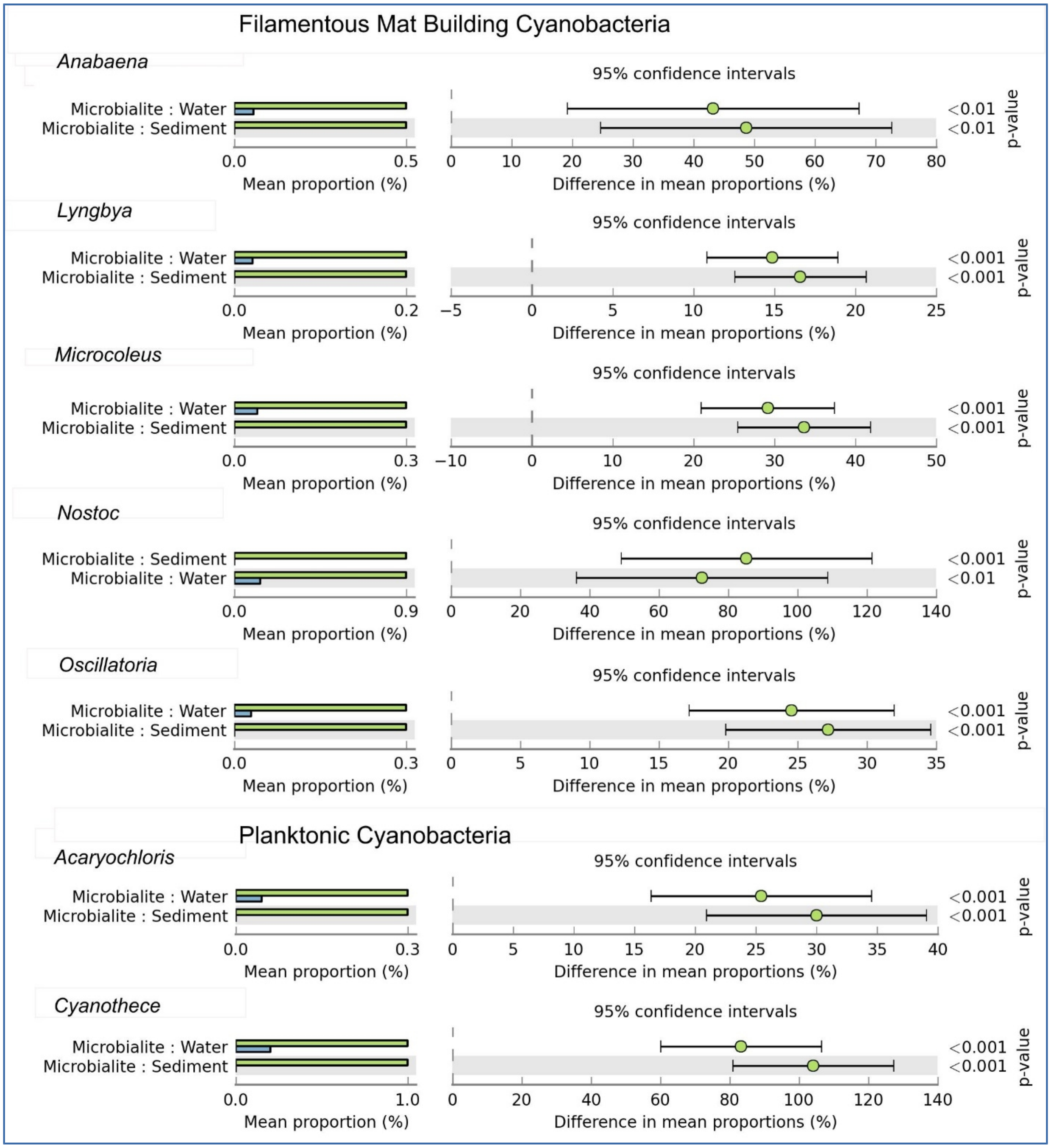

Figure S6

Post-havoc confidence interval plots for *Cyanobacteria* using RefSeq classification.
Based on ANOVA in STAMP for significant genus-level members represented in Pavilion Lake metagenomes (microbialite, water filter and sediment) using multiple groups.
Microbialites are labeled in green, followed by sediments in orange and water in blue.

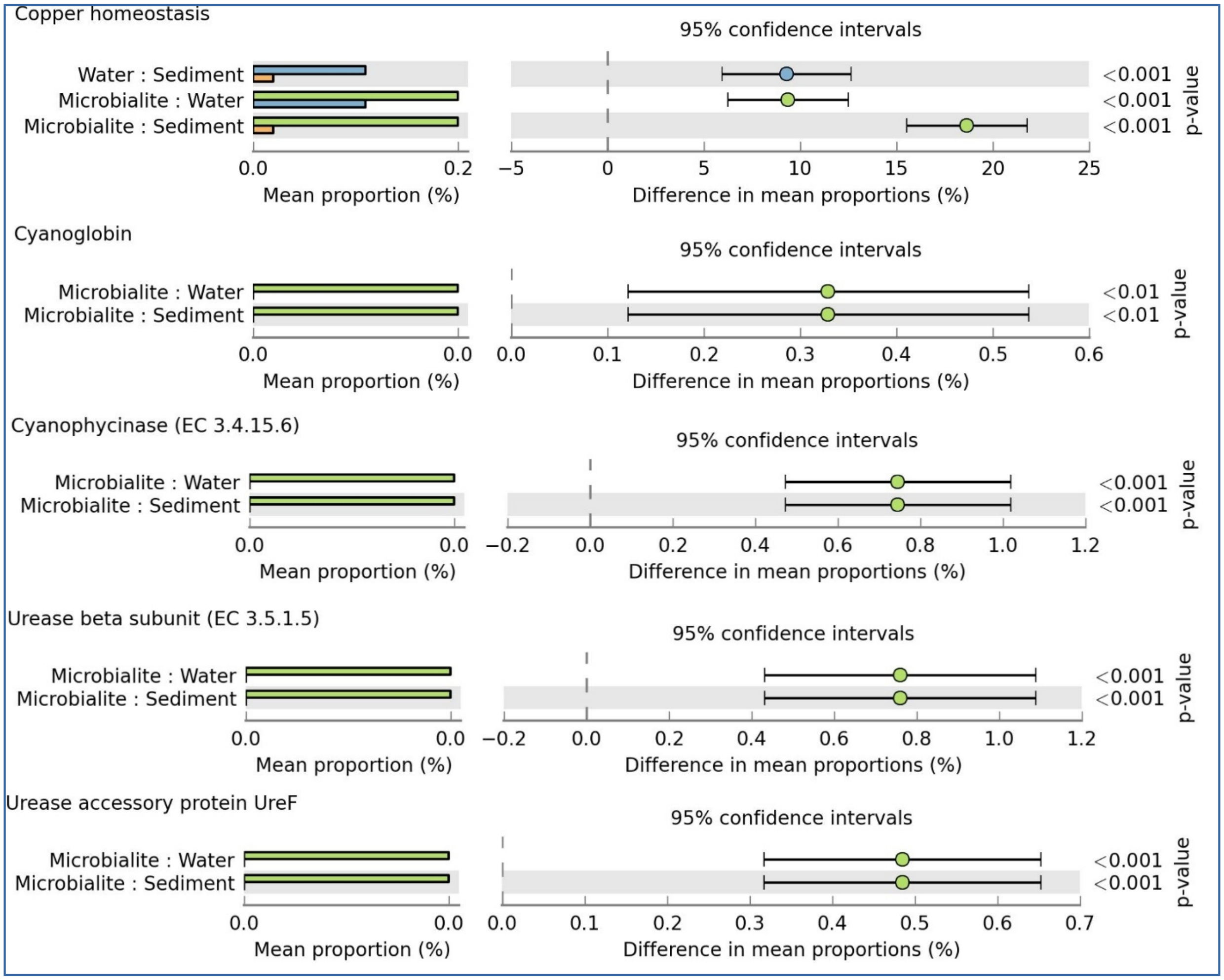

Figure S7

Post-havoc confidence interval plots for cyanobacterial based functional genes using SEED subsystem classification and urealytic pathways

Based on ANOVA in STAMP for significant functional classifications represented in Pavilion Lake metagenomes (microbialite, water filter and sediment) using multiple groups.

The urealytic genes are for general presence in the metagenomes and are not cyanobacterial specific. Microbialites are labeled in green, followed by sediments in orange and water in blue.

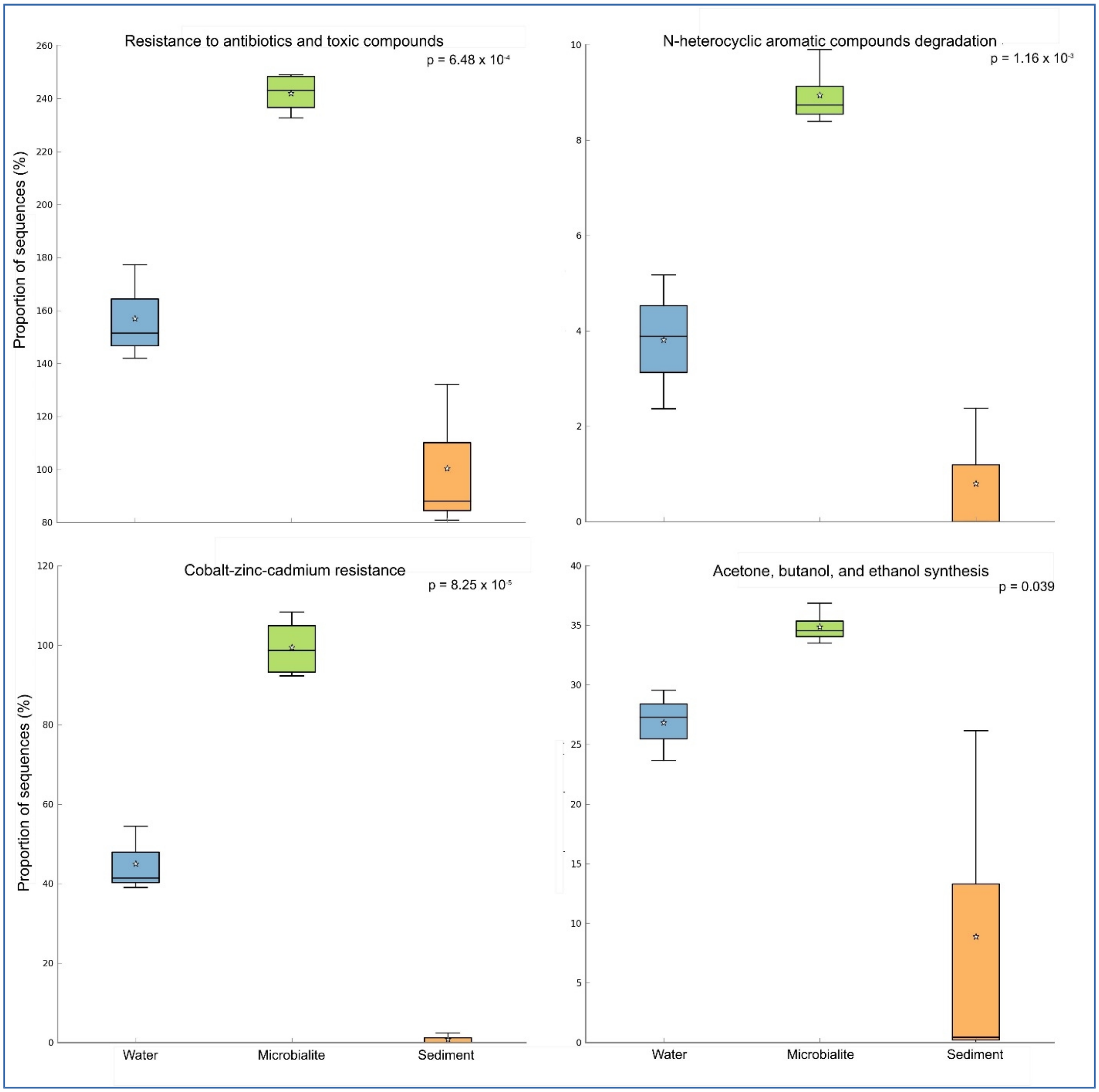

Figure S8

Box and whisker plots for novel functional classifications (SEED subsystem) represented in microbialites based on ANOVA in STAMP using multiple groups.

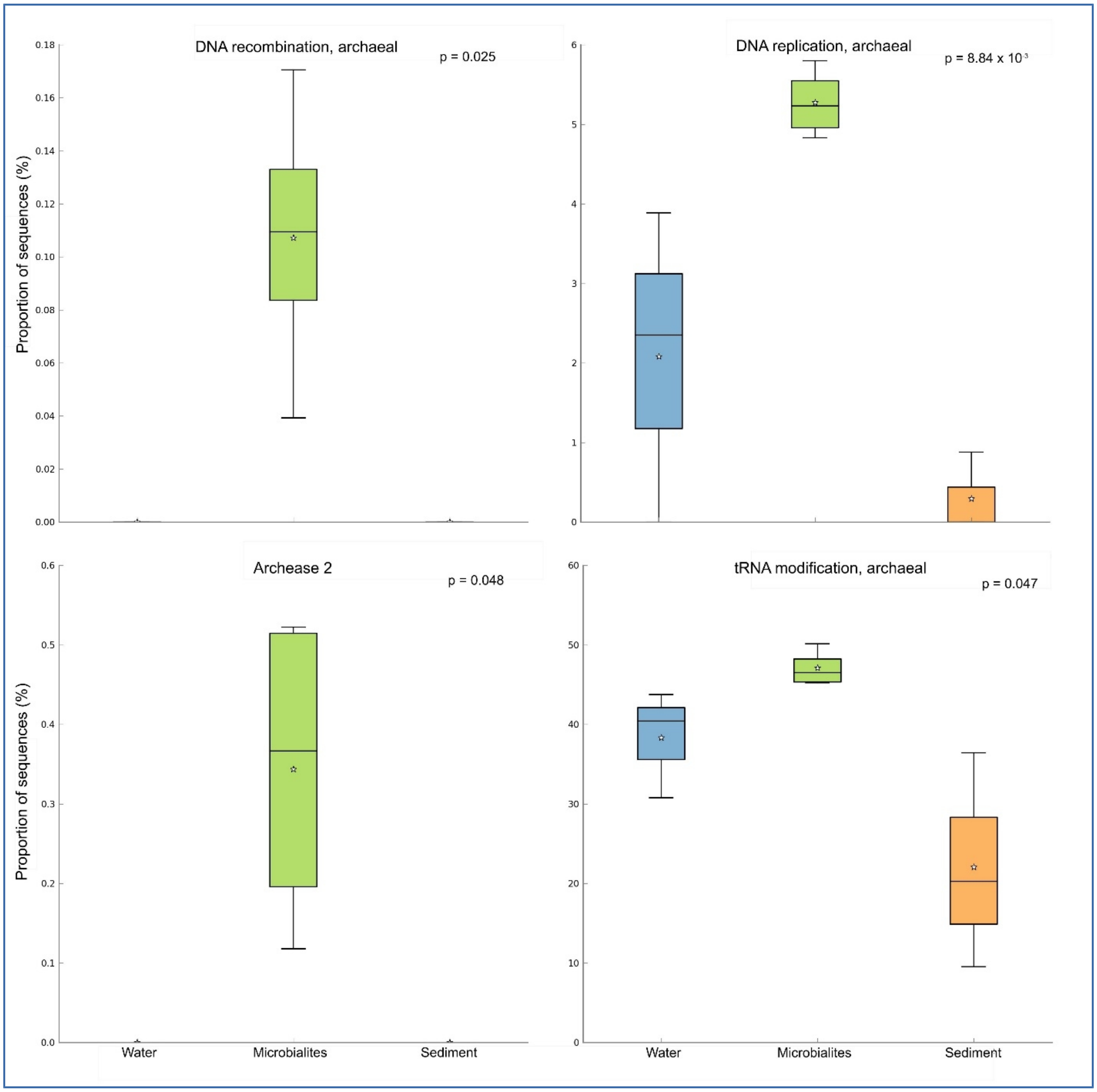

Figure S9

Box and whisker plots for novel Archael based functional classifications (SEED subsystem) represented in microbialites based ANOVA in STAMP using multiple groups

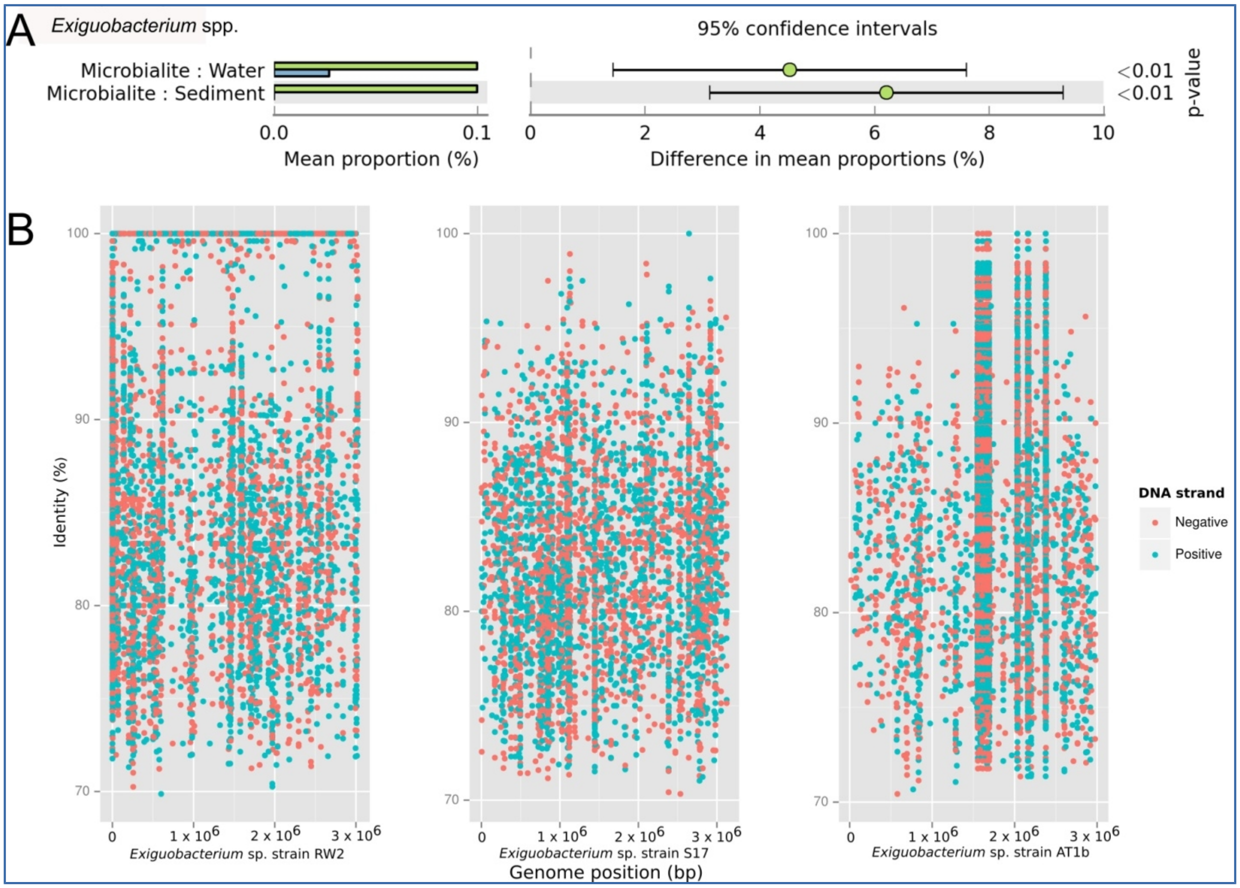

Figure S10

Exiguobacterium presence in Pavilion Lake microbialite metagenome.

A) *Exiguobacterium* post-havoc confidence interval plots (0.95) based on ANOVA parameters for novel metabolic potential differences for microbialites and surrounding environment using multiple groups in STAMP.

B) Metagenomic recruitment plot of Pavilion lake 20 m microbialite reads (~7.5 Million) to three *Exiguobacterium* genetically related to *Exiguobacterium* sp. strain RW2 (minimum identity >70% and an E-value >1e^{-5}).

**Exiguobacterium* sp. strain RW2 was isolated from a microbialite at 20 m (White 3rd *et al.,* 2013b).

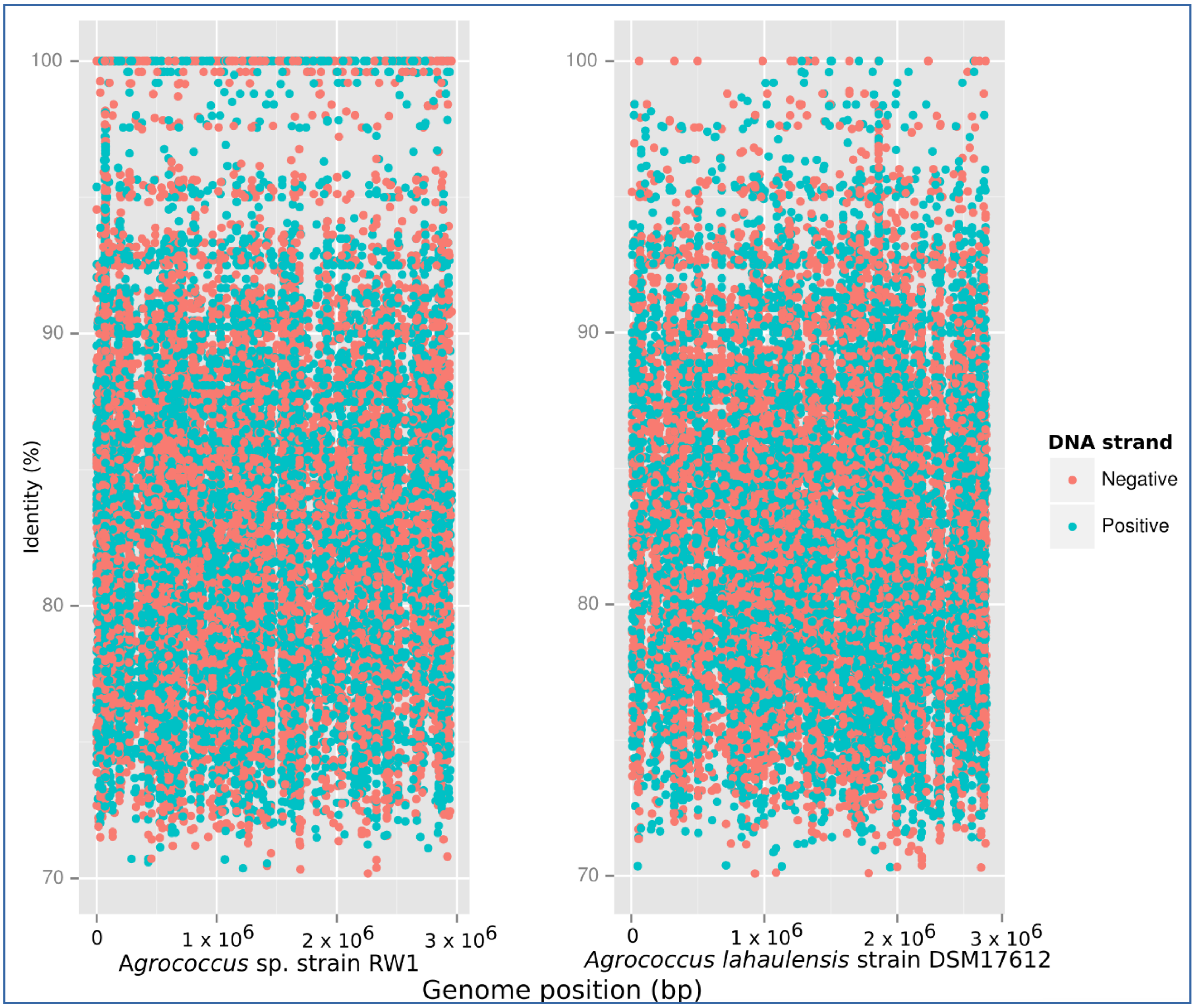

Figure S11
Metagenomic recruitment plot for Pavilion Lake microbialite against *Agrococcus*
FR-Hit recruitment plot of Pavilion lake 20 m microbialite reads (~7.5 Million) to *Agrococcus* sp. strain RW1 and to *Agrococcus lahaulensis* strain DSM17612 (Minimum identity >70% and an E-value >1e^{-5}).
**Agro*coccus sp. strain RW1 was isolated from a microbialite at 20 m (White 3rd *et al.,* 2013a).

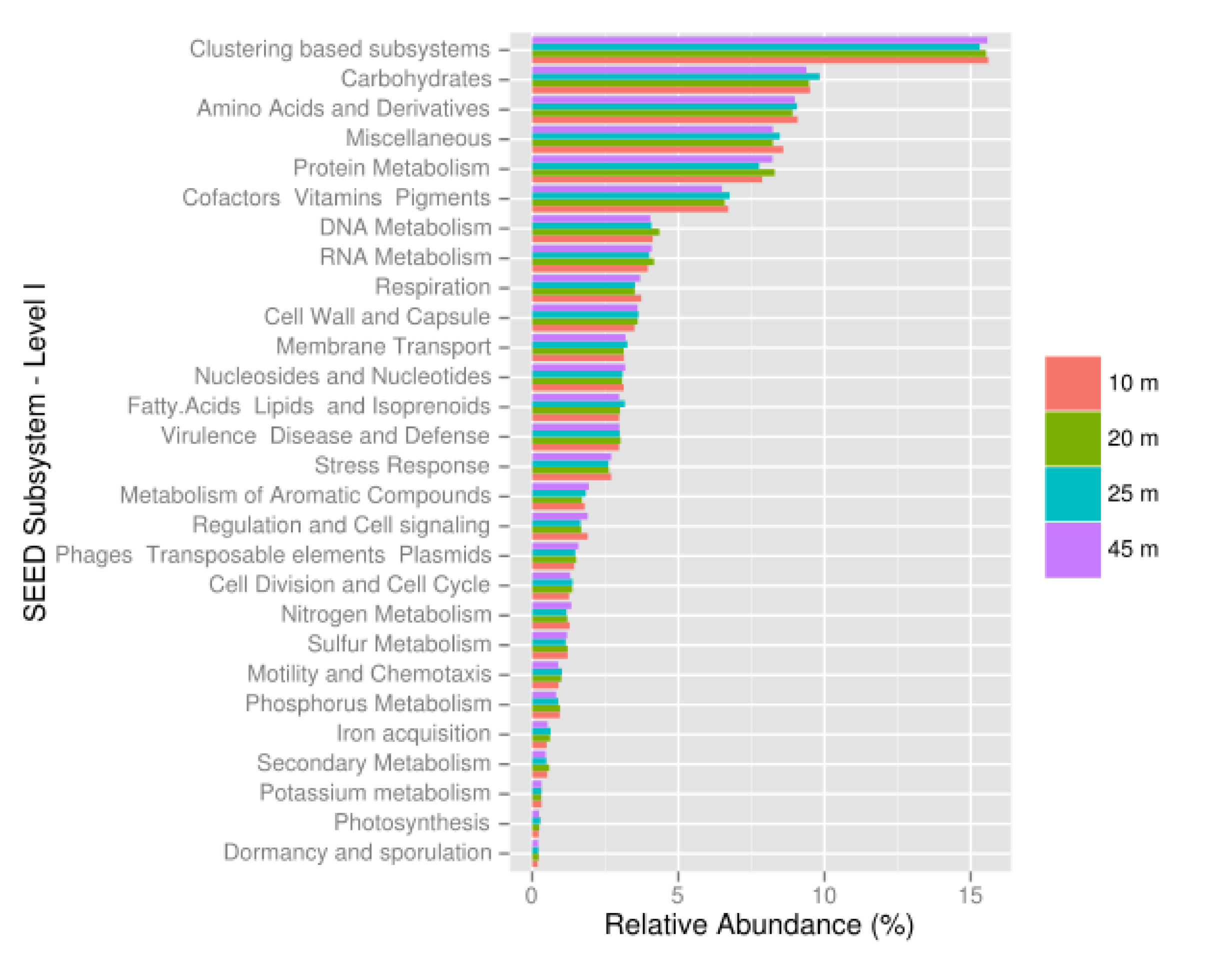

Figure S12

Microbialite metagenome functional annotation ranking using SEED subsystem (Level I) Microbialite metagenomes are listed as a function of depth (m).

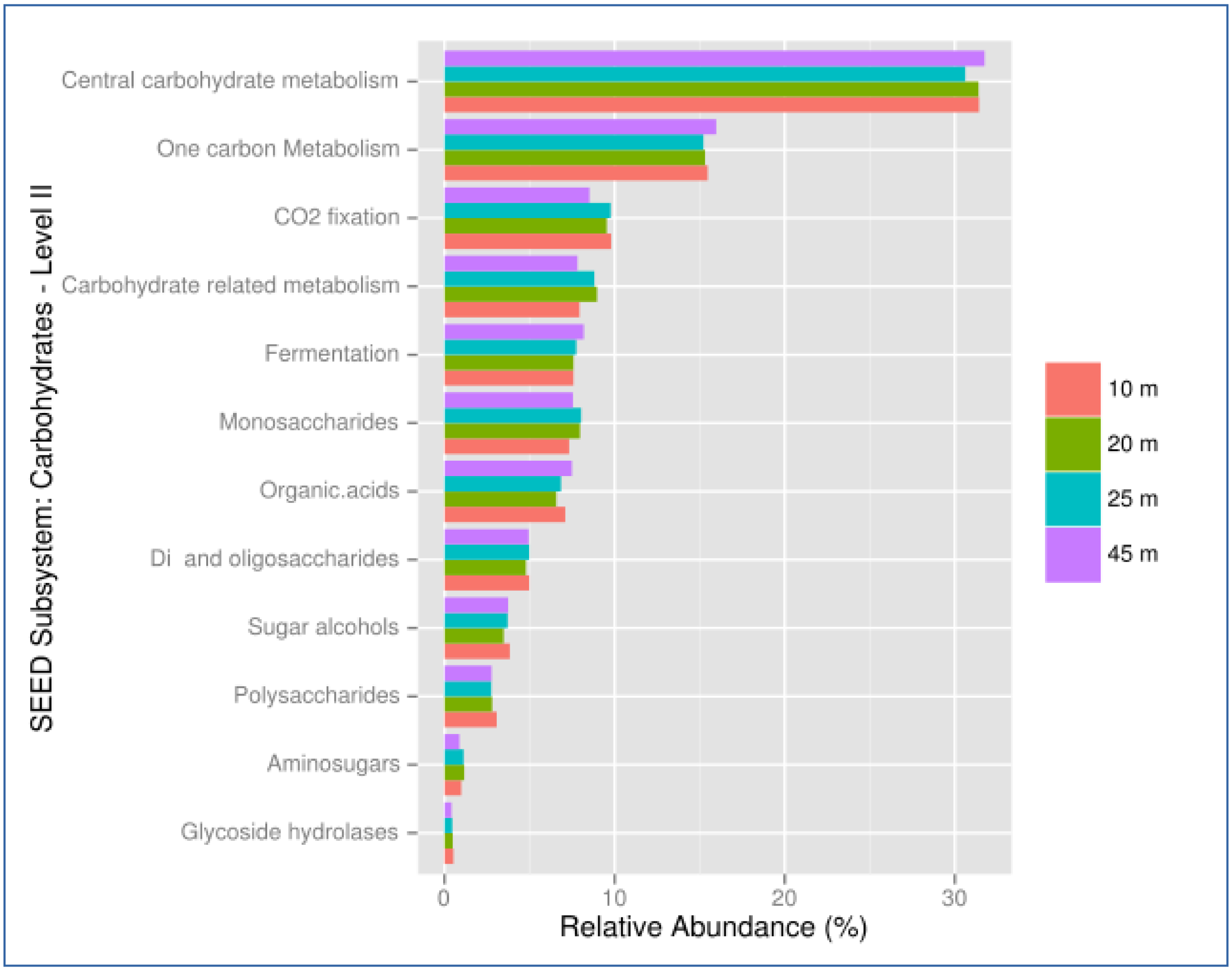

Figure S13
Microbialite metagenome carbohydrate related SEED subsystem (Level II) functional annotations. Microbialite metagenomes are listed as a function of depth (m).

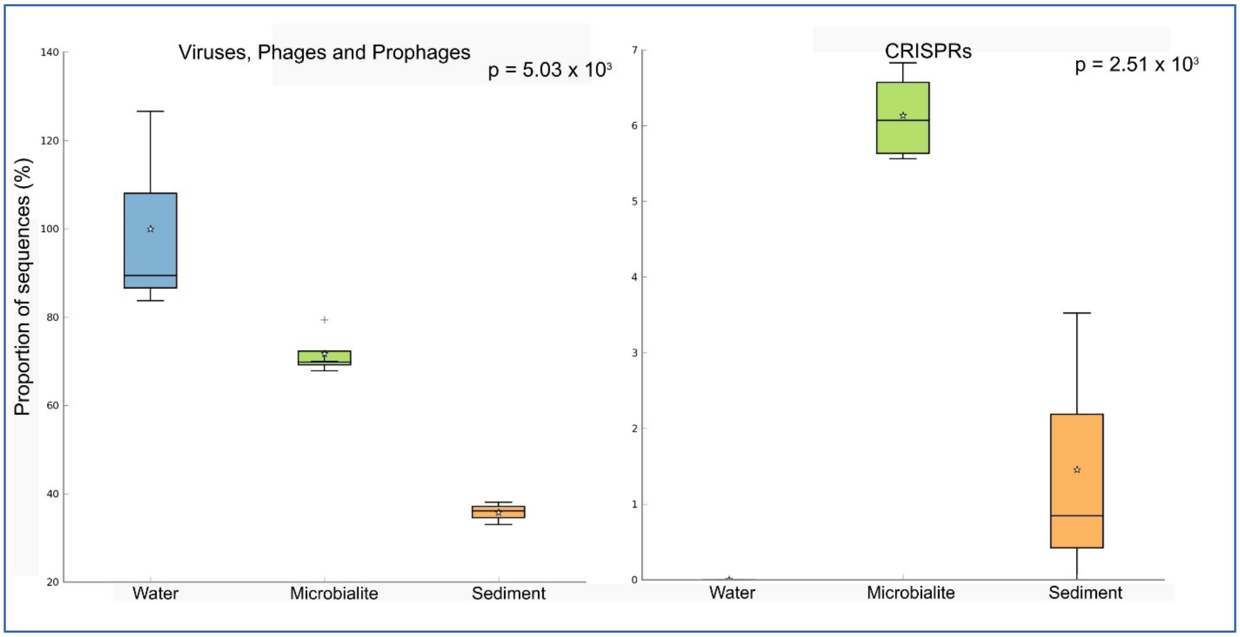

Figure S14

Box and whisker plots for viruses and CRISPRs in Pavilion Lake.

RefSeq and SEED subsystem represented in microbialites based on ANOVA in STAMP using multiple groups

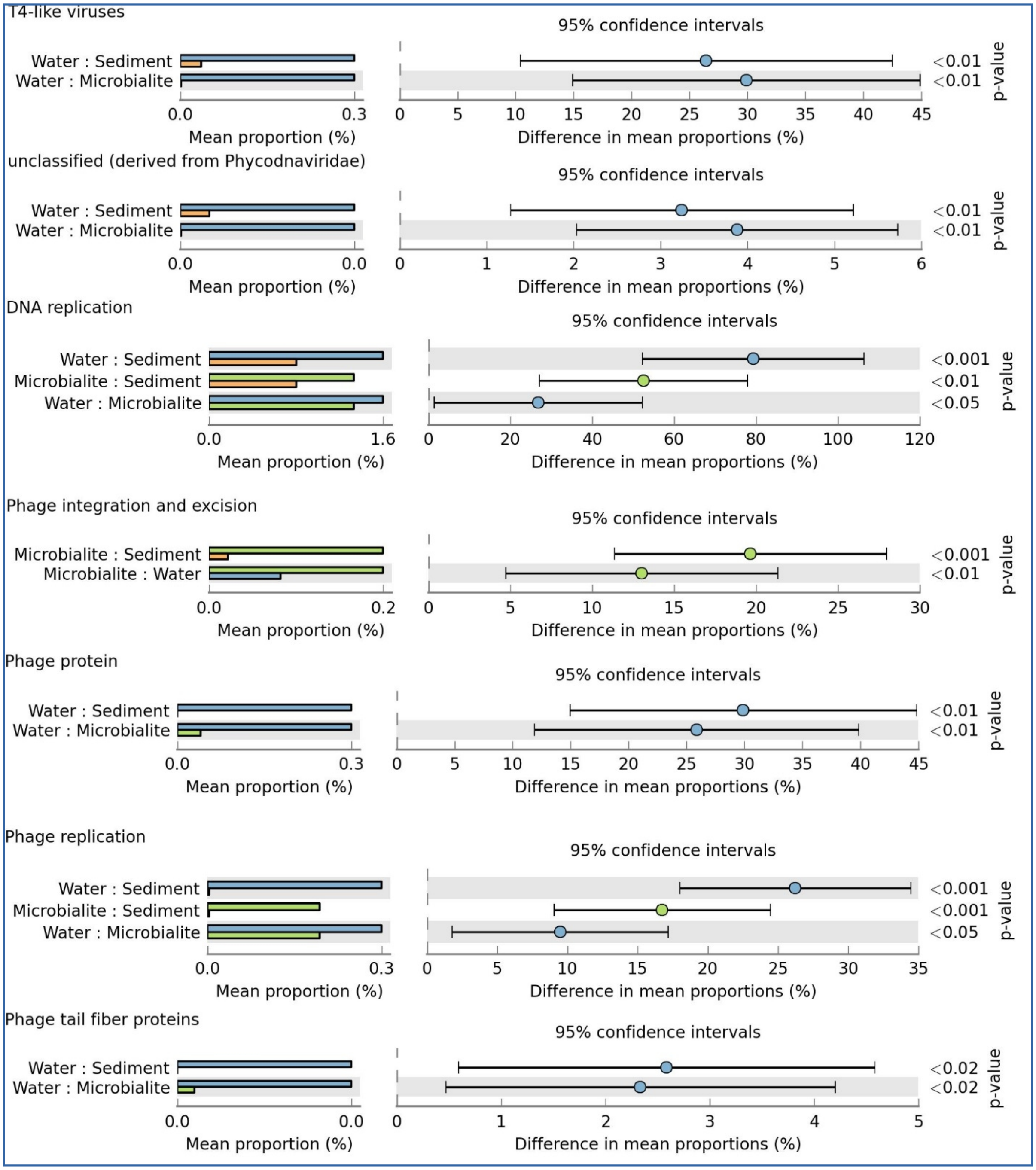

Figure S15

Post-havoc confidence interval plots for viral taxa using RefSeq and viral specific functional genes in SEED. Based on ANOVA in STAMP for genus-level and functional gene-level classifications that were statistically significant by analysis using multiple groups.
